# Multiple Holdouts With Stability: Improving the Generalizability of Machine Learning Analyses of Brain–Behavior Relationships

**DOI:** 10.1016/j.biopsych.2019.12.001

**Published:** 2020-02-15

**Authors:** Agoston Mihalik, Fabio S. Ferreira, Michael Moutoussis, Gabriel Ziegler, Rick A. Adams, Maria J. Rosa, Gita Prabhu, Leticia de Oliveira, Mirtes Pereira, Edward T. Bullmore, Peter Fonagy, Ian M. Goodyer, Peter B. Jones, NeuroScience in Psychiatry Network (NSPN) Consortium, Tobias Hauser, Sharon Neufeld, Rafael Romero-Garcia, Michelle St Clair, Petra E. Vértes, Kirstie Whitaker, Becky Inkster, Cinly Ooi, Umar Toseeb, Barry Widmer, Junaid Bhatti, Laura Villis, Ayesha Alrumaithi, Sarah Birt, Aislinn Bowler, Kalia Cleridou, Hina Dadabhoy, Emma Davies, Ashlyn Firkins, Sian Granville, Elizabeth Harding, Alexandra Hopkins, Daniel Isaacs, Janchai King, Danae Kokorikou, Christina Maurice, Cleo McIntosh, Jessica Memarzia, Harriet Mills, Ciara O’Donnell, Sara Pantaleone, Jenny Scott, Pasco Fearon, John Suckling, Anne-Laura van Harmelen, Rogier Kievit, John Shawe-Taylor, Raymond Dolan, Janaina Mourão-Miranda

**Affiliations:** a Centre for Medical Image Computing, Department of Computer Science, University College London, London, United Kingdom; b Max Planck University College London Centre for Computational Psychiatry and Ageing Research, University College London, London, United Kingdom; c Wellcome Centre for Human Neuroimaging, University College London, London, United Kingdom; d Research Department of Clinical, Educational, and Health Psychology, University College London, London, United Kingdom; e Department of Computer Science, University College London, London, United Kingdom; f Department of Psychiatry, University of Cambridge, Cambridge, United Kingdom; g Behavioural and Clinical Neuroscience Institute, University of Cambridge, Cambridge, United Kingdom; h Cambridgeshire and Peterborough NHS Foundation Trust, Cambridge, United Kingdom; i ImmunoPsychiatry, GlaxoSmithKline Research and Development, Stevenage, United Kingdom; j Institute of Cognitive Neurology and Dementia Research, Otto von Guericke University, Magdeburg, Magdeburg, Germany; k German Center for Neurodegenerative Diseases, Bonn, Germany; l Laboratory of Neurophysiology of Behaviour, Department of Physiology and Pharmacology, Biomedical Institute, Federal Fluminense University, Niterói, Brazil

**Keywords:** Adolescence, Brain–behavior relationship, Depression, Framework, RDoC, SPLS

## Abstract

**Background:**

In 2009, the National Institute of Mental Health launched the Research Domain Criteria, an attempt to move beyond diagnostic categories and ground psychiatry within neurobiological constructs that combine different levels of measures (e.g., brain imaging and behavior). Statistical methods that can integrate such multimodal data, however, are often vulnerable to overfitting, poor generalization, and difficulties in interpreting the results.

**Methods:**

We propose an innovative machine learning framework combining multiple holdouts and a stability criterion with regularized multivariate techniques, such as sparse partial least squares and kernel canonical correlation analysis, for identifying hidden dimensions of cross-modality relationships. To illustrate the approach, we investigated structural brain–behavior associations in an extensively phenotyped developmental sample of 345 participants (312 healthy and 33 with clinical depression). The brain data consisted of whole-brain voxel-based gray matter volumes, and the behavioral data included item-level self-report questionnaires and IQ and demographic measures.

**Results:**

Both sparse partial least squares and kernel canonical correlation analysis captured two hidden dimensions of brain–behavior relationships: one related to age and drinking and the other one related to depression. The applied machine learning framework indicates that these results are stable and generalize well to new data. Indeed, the identified brain–behavior associations are in agreement with previous findings in the literature concerning age, alcohol use, and depression-related changes in brain volume.

**Conclusions:**

Multivariate techniques (such as sparse partial least squares and kernel canonical correlation analysis) embedded in our novel framework are promising tools to link behavior and/or symptoms to neurobiology and thus have great potential to contribute to a biologically grounded definition of psychiatric disorders.

Psychiatric diagnoses [e.g., DSM-5 (1), ICD-10 (2)] lack neurobiological validity (3, 4, 5). To address this, the National Institute of Mental Health launched Research Domain Criteria (RDoC) (6) in 2009, a research framework that “integrates many levels of information (from genomics and circuits to behavior) in order to explore basic dimensions of functioning that span the full range of human behaviour from normal to abnormal” (https://www.nimh.nih.gov/research-priorities/rdoc/index.shtml). RDoC represents a paradigm shift in psychiatry and highlights the need to include measures of genes, brain, and behavior to understand psychopathology (4,6). RDoC is structured as a matrix with 4 dimensions: 1) domains of functioning (e.g., negative–positive valence systems) that are further divided into constructs (e.g., attention, perception), 2) units of analysis (e.g., genes, circuits, behavior), 3) developmental aspects, and 4) environmental aspects. Analyzing data containing multiple such modalities, however, poses statistical challenges. Here, we propose a novel framework that is robust to some typical problems arising from high-dimensional neurobiological data such as overfitting, poor generalization, and interpretability of the results.

Factor analysis and related methods (e.g., principal component analysis [PCA]) have long traditions in statistics and psychology (7,8). These techniques decompose a single set of measures (e.g., self-report questionnaires) into a parsimonious, latent dimensional representation of the data. Applications of these approaches include general intelligence [*g* factor (8)], the five-factor personality model (9), and many others (10, 11, 12, 13). However, factor analysis cannot integrate different sets of measures/modalities (e.g., investigate brain–behavior relationships).

A principled way to find latent dimensions of one modality (or data type) that is related to another modality (or data type) is to use partial least squares (PLS) (14) or the closely related canonical correlation analysis (CCA) (15). PLS was introduced to neuroimaging by McIntosh *et al.* (16), and it has been widely used (17, 18, 19, 20, 21, 22). Unfortunately, the high dimensionality of neuroimaging data makes PLS and CCA models prone to overfitting; moreover, the interpretation of the identified latent dimensions is usually difficult. Regularized versions of PLS and CCA algorithms address these issues (23, 24, 25); two popular choices are lasso (26) and elastic net (27) regularization, which constrain the optimization problem to select the most relevant variables.

Sparse CCA and sparse PLS (SPLS) were originally proposed in genetics (23,28, 29, 30) and have since been used in cognition (31, 32, 33, 34), working memory (35,36), dementia (37, 38, 39, 40, 41), psychopathology in adolescents (42), psychotic disorders (43, 44, 45, 46), and pharmacological interventions (47). However, most of these studies used approaches for selecting the regularization parameter (model selection) and inferring statistical significance of the identified relationships (model evaluation) that do not account for the generalizability and stability of the results.

Here, we propose an innovative framework combining stability and generalizability as optimization criteria in a multiple holdout framework (48) that is applicable to both regularized PLS and CCA approaches. Crucially, it increases the reproducibility and generalizability of these models by 1) applying stability/reproducibility for model selection and 2) using out-of-sample correlations of the data for model evaluation. To demonstrate this novel framework, we investigated associations between whole-brain voxel-based gray matter volumes and item-level measures of self-report questionnaires, IQ, and demographics in a sample of healthy adolescents and young adults (*n* = 312) and adolescents and young adults with depression (*n* = 33). We report the results from using SPLS in the main text, and for comparison we include results with another regularized approach, kernel CCA (KCCA), in the Supplement.

## Methods and Materials

### PLS/CCA and Other Latent Variable Models

**Figure 1:**
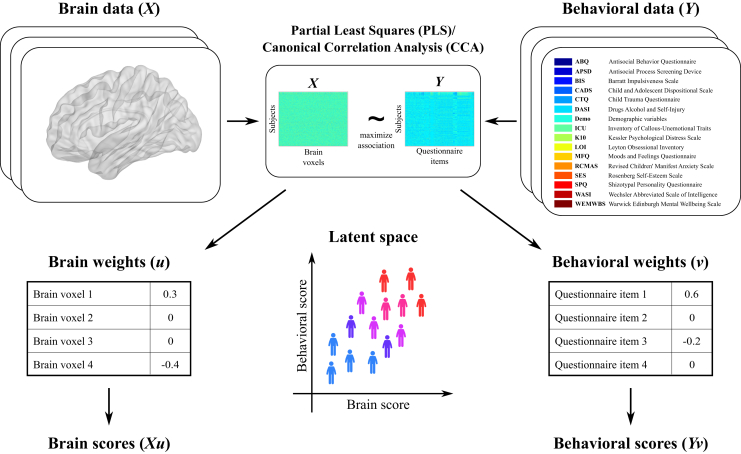
Overview of the partial least squares/canonical correlation analysis (PLS/CCA) models. PLS/CCA models search for weight vectors that maximize the covariance (PLS) or correlation (CCA) between linear combinations of the brain and behavioral variables. Importantly, the sparsity constraints of sparse PLS set some of the brain and behavioral weights to zero. The linear combination (i.e., weighted sum) of brain and behavioral variables (columns of *X* and *Y*) with the respective weights (elements of *u* and *v*) results in brain and behavioral scores (*Xu* and *Yv*) for each individual subject. The brain and behavioral scores can be combined to create a brain–behavior latent space showing how the brain–behavior relationship (i.e., association) is expressed across the whole sample.

Figure 1 illustrates how PLS/CCA models can be used to identify latent dimensions of brain–behavior relationships. PLS/CCA maximizes the association (covariation for PLS and correlation for CCA) between linear combinations of brain and behavioral variables. The model’s inputs are brain and behavioral variables for multiple subjects (e.g., voxel-level gray matter volumes and item-level questionnaires). Its outputs, for each brain–behavior relationship, are brain and behavioral weights, brain and behavioral scores, and a value denoting the strength of the correlation/covariation.

The brain and behavioral weights have the same dimensionality as their respective data and quantify each brain and behavioral variable’s contribution to the identified brain–behavior relationship or association. Once the weights are found, then brain and behavioral scores can be computed for each subject as a linear combination (i.e., weighted sum) of their brain and behavioral variables, respectively. The brain and behavioral scores can then be combined to create a latent space of brain–behavior relationships across the sample. Furthermore, each brain–behavior relationship can be removed from the data (by a process called deflation) and new relationships sought.

Next, we present a brief overview of PLS/CCA and some other latent variable models to contextualize our modeling approaches. Essentially, all these models search for weight vectors or directions, such that the projection of the dataset(s) (e.g., the brain and/or behavior) onto the obtained weight vector(s) has maximal variance (PCA), correlation (CCA), or covariance (PLS) (49, 50, 51). Note that PCA is limited to finding latent dimensions in one dataset (e.g., behavior). Although its principal components can be used in a multiple regression (referred to as principal component regression), such as to predict brain variables, the directions of high variance identified by PCA might be uncorrelated with the brain variables, while a relatively low variance component might be a useful predictor. Therefore, CCA and PLS can be seen as extensions of principal component regression to find latent dimensions relating two sets of data to each other (50,52).

In the regularized versions of CCA/PLS, additional constraints (governed by regularization parameters) are added to the optimization problem to control the complexity of the CCA/PLS model and reduce overfitting. A regularized version of CCA was proposed by Hardoon *et al.* (53), in which two regularization parameters control a smooth transition between maximizing correlation (i.e., a CCA-like least-regularized solution) and maximizing covariance (i.e., a PLS-like most-regularized solution). Our KCCA implementation is an extension of this regularized CCA, where the kernel formulation makes the algorithm computationally more efficient (50).

A regularized sparse version of CCA was proposed by Witten *et al.* (30), which applies elastic net regularization to the weight vectors. Interestingly, because the variance matrices are assumed to be identity matrices in this optimization, their formulation becomes equivalent to our SPLS implementation. Elastic net regularization combines the L1 and L2 constraints of the lasso and ridge methods, respectively. The L1 constraint shrinks some weights and sets others to zero, leading to automatic variable selection (26); however, it has 3 main limitations: 1) selecting at most as many variables as the number of examples/samples in the data, 2) selecting only a few from correlated groups of variables, and 3) leading to worse prediction than ridge regression when the variables are highly correlated (27). The L2 constraint shrinks the weights but does not set them to zero, enabling correlated variables to have similar weights. Combining both the L1 and L2 constraints, elastic net regularization can simultaneously enforce sparse solutions and select correlated variables while enabling optimal prediction performance (27). For further details of PLS/CCA models, see the Supplement.

### Model Selection and Statistical Evaluation

To motivate our proposed framework, we briefly review two landmark SPLS applications and their methods of model selection (i.e., regularization parameter choice) and statistical inference.

In one of the most popular SPLS applications, Witten *et al.* (30) proposed 2 approaches: 1) fixing the regularization parameters of the data a priori and performing permutation testing for model evaluation and 2) using the same permutation test for both selecting the regularization parameters and evaluating the model. In the permutation test, the SPLS model is fitted to the original datasets and to the permuted datasets (i.e., after randomly shuffling one of the datasets); *p* values are calculated by comparing the SPLS model correlations from the original and the permuted (null) data. When this framework is also used for selecting the regularization parameters, the same procedure is repeated for each combination of regularization parameters (there is one regularization parameter for each dataset, e.g., brain and behavior), and the combination of values leading to the smallest *p* value is selected. Many other studies followed similar approaches either fixing the regularization parameters (23,32,38,41,54) or choosing them based on permutation tests (55,56). This framework might be preferable when the sample size is small; however, because it does not test whether the identified association generalizes to unseen or holdout data, this approach might overfit the data.

**Figure 2:**
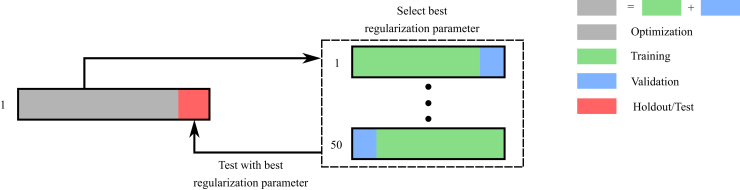
Multiple holdout framework. The original data are randomly split to an optimization set (80% of the data) and a holdout set (20% of the data). The optimization set is used to fit the regularized partial least squares/canonical correlation analysis model and optimize the regularization parameters in 50 further training and validation splits. The best regularization parameter is used to fit the regularized partial least squares/canonical correlation analysis model on the whole optimization set, and the resulting model is evaluated on the holdout set using permutation testing. Finally, the entire procedure is repeated 10 times.

Monteiro *et al.* (48) proposed a multiple holdout framework to optimize the regularization parameters and test the generalizability of the optimized SPLS models (Figure 2). This framework fits the SPLS model on an optimization set (e.g., 80% of the data) and assesses the identified multivariate associations on a holdout set (e.g., 20% of the data). The regularization parameters are selected by further splitting the optimization set into training and validation sets and choosing the combination of parameters with better generalization performance (measured by the out-of-sample correlation) on the validation set. To further test the robustness of the SPLS model, the entire procedure is repeated 10 times. This framework goes beyond many other SPLS approaches, which split the data once (or use cross-validation) to select the regularization parameters but do not evaluate the model generalizability on an independent test or holdout set (28,33,42,57). Although this framework provides a good test of model generalizability, it does not account for stability of the models across the different data splits while selecting the regularization parameters.

Our proposed framework is similar to that of Monteiro *et al.* (48), but it performs regularization parameter selection using stability and generalizability as a joint optimization criterion, extending the work of Baldassarre *et al.* (58) to regularized PLS/CCA models (Supplemental Figure S1). We measure generalizability as the average out-of-sample correlation on the validation and holdout sets for selecting regularization parameter and model evaluation, respectively. Stability is measured by the average similarity of weights (corrected overlap for SPLS and absolute correlation for KCCA) across splits, that is, how often the models (trained on different subsets of the data) select similar brain and behavioral variables (see Supplement). This joint criterion for parameter selection should enable the identification of brain–behavior associations that are stable and can generalize well to new data.

### Data

A total of 345 participants from the NeuroScience in Psychiatry Network (NSPN) project (59) were included in this study (312 healthy participants, mean age = 19.14 ± 2.93 years, 156 female; 33 participants with depression, mean age = 16.50 ± 1.23 years, 23 female). See the Supplement for the details of data acquisition and processing.

All participants completed an abbreviated IQ test and extensive self-report questionnaires assessing well-being, affective symptoms, anxiety, impulsivity and compulsivity, self-esteem, self-harm, antisocial and callous-unemotional characteristics, psychosis spectrum symptoms, substance use, relations with peers and family, and experience of trauma. We added 3 demographic variables (age, sex, and socioeconomic status index) to the items of these questionnaires, resulting in a total of 364 variables, which we call behavioral data for simplicity. Including these demographic variables explicitly in the SPLS model permits investigation of whether these variables interact with brain–behavior relationships. Structural imaging scans were acquired on identical 3T Siemens Magnetom Tim Trio systems (Siemens, Erlangen, Germany) across 3 sites. Only scans at the baseline study visit were included in the current analysis. Structural scans (∼19 minutes) were acquired using a quantitative multiparameter mapping protocol (60). Structural magnetic resonance imaging data preprocessing was performed using SPM12 (https://www.fil.ion.ucl.ac.uk/spm), including segmentation, normalization, downsampling, and smoothing (see Supplement). We then applied a mask selecting voxels with ≥10% probability of containing gray matter to all participants, resulting in a total of 219,079 voxels (brain data). Two confounds were removed (i.e., regressed out) from both datasets: total intracranial volume and data collection site (17,61).

## Results

SPLS identified 2 significant latent dimensions of brain–behavior associations in our sample. Because the proposed framework fits the model to different splits of the data, here we present the results for the split that presented the best combination of generalizability (measured by the out-of-sample correlation on the holdout set) and stability (measured by the similarity of weights across the optimization sets). Results for the other data splits are in Supplemental Tables S1–S3 and Supplemental Figures S2 and S3.

**Figure 3:**
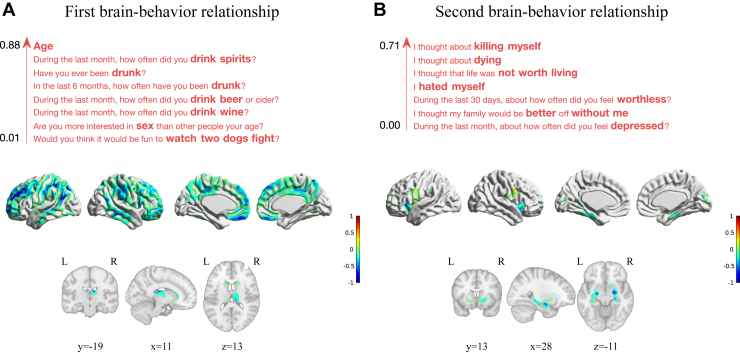
Brain and behavioral weights of the two significant associative brain–behavior relationships identified by sparse partial least squares. The brain voxels are color coded by weight, normalized for visualization purposes, and displayed on Montreal Neurological Institute 152 template separately for subcortical (including hippocampus) and cortical regions. The behavioral variables are ordered by weight and color coded with red for positive weights. **(A)** Brain and behavioral weights of the first brain–behavior relationship. **(B)** Brain and behavioral weights of the second brain–behavior relationship. L, left; R, right.

The first brain–behavior relationship (*p* = .001) captured an association between age and drinking habits and a widespread set of frontoparietotemporal cortical regions, including the medial wall (middle and posterior cingulate and medial orbital cortices), inferior parietal cortex, orbitofrontal cortex, dorsolateral prefrontal cortex, right inferior frontal gyrus, and middle temporal gyri (Figure 3). The brain weights are further summarized using an anatomical atlas in Supplemental Table S4. As expected, the SPLS weights were sparse, selecting 2% of the behavioral variables (1.73 ± 0.48% SEM across data splits) and 22% of the brain variables (36.75 ± 4.62% SEM across data splits).

The second brain–behavior relationship (*p* = .014) captured an association between behavioral items related to depression, self-harm, and gray matter volume in a small set of regions, including the hippocampus, parahippocampal gyrus, insula, amygdala, pallidum, and putamen (Figure 3). The behavioral variables related to depression included items such as “feeling worthless,” “hated myself,” and “feeling depressed,” and the behavioral items related to self-harm included thinking about “killing myself” and “thought about dying.” The brain weights are further summarized using an anatomical atlas in Supplemental Table S4. Again, SPLS resulted in rather sparse weights, selecting 2% of the behavioral variables (3.35 ± 1.13% SEM across data splits) and 5% of the brain variables (11.85 ± 3.15% SEM across data splits).

**Figure 4:**
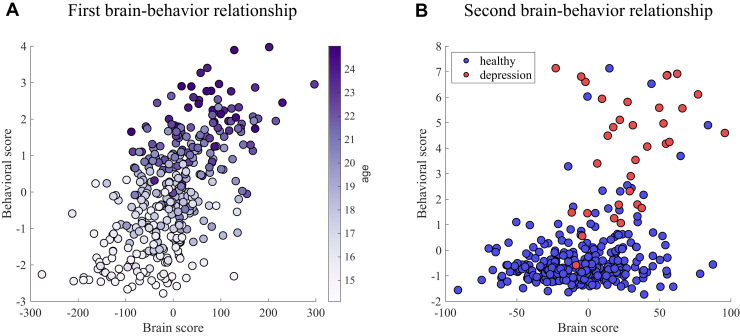
Two significant brain–behavior latent spaces identified by sparse partial least squares. **(A)** Scatterplot of the brain and behavioral scores of the first brain–behavior relationship with subjects color coded by age. **(B)** Scatterplot of the brain and behavioral scores of the second brain–behavior relationship with subjects color coded by clinical diagnosis.

Scatterplots of the brain and behavioral scores allow us to examine how the brain–behavior relationship is expressed across the whole sample (Figure 4). The first multivariate associative effect clearly maps to age, while the second multivariate effect captured a brain–behavior association that varied from healthy to depressed, with subjects with depression presenting higher brain and behavioral scores.

For comparison, we performed 2 additional analyses. First, we added age to the confounds in the SPLS analysis to discount any sampling bias given that the subjects with depression were younger. Here, we identified 1 significant brain–behavior relationship that was very similar to the second depression-related associative effect of the main analysis (*p* = .047) (Supplemental Figures S4 and S5 and Supplemental Tables S5 and S6). Second, we used KCCA to demonstrate the framework with an alternative regularized approach. Here, we identified 2 significant brain–behavior relationships that were very similar to those identified by SPLS (first associative effect: *p* = .001 [Supplemental Figures S6A and S7 and Supplemental Tables S7 and S8]; second associative effect: *p* = .006 [Supplemental Figures S6B and S8 and Supplemental Tables S7 and S8]). For a detailed description of these results, see the Supplemental Results.

## Discussion

We presented a novel framework combining stability and generalizability as optimization criteria for regularized multivariate methods, such as SPLS and KCCA, which decreases their risk of detecting spurious associations, particularly in high-dimensional data. Furthermore, we demonstrated that this framework can identify brain–behavior relationships that capture developmental variation as well as variations from normal to abnormal functioning.

Our proposed framework coheres with the overarching intentions of RDoC (62,63). First, SPLS and KCCA link different levels of measures in a principled integrated analysis: here, the key levels are circuits and physiology (in brain imaging) and behavior and self-report. Indeed, RDoC views circuits as the key level anchoring and integrating the rest; however, without robust multivariate techniques, it is challenging to relate circuits to behavior in large-scale human datasets. Second, the latent dimensions identified by SPLS and KCCA may be fundamental axes of neurobiological variation spanning healthy to abnormal functioning. Application of this framework to sufficiently large clinical samples therefore might yield domains of mental (dys)function that are driven by data rather than chosen by experts (as in RDoC itself). Third, the SPLS and KCCA models output brain and behavioral scores for each individual subject in the identified latent dimensions; this is a crucial step toward using RDoC (or similar approaches) for clinical diagnosis.

The model selection and statistical inference in our framework differs from those of other SPLS approaches in the literature. (Note also that these methods are not limited to SPLS but are also relevant for any regularized PLS/CCA models, including KCCA.) For model selection, some suggest fixing the regularization parameters a priori (23,30,32,38,41,54) or choosing the regularization parameters based on the performance of the SPLS model (e.g., maximizing the correlation or the associated *p* value of the obtained model) (36,45,46,56). Our framework is similar to other data-driven approaches that split the data into training and validation sets and use the validation set to evaluate the SPLS model and select the optimal regularization parameters (28,43,57). For model evaluation, most studies use permutation testing to evaluate the SPLS model based on all available data (30,32,45,55,56); however, this approach does not assess the model’s generalization to new data. To perform statistical inference on how the SPLS model generalizes to unseen data, independent test data (or holdout set) are needed to evaluate it (e.g., Figure 2) [as used, for example, in (23,39)]. If a validation set is used to select the optimal regularization parameter, 3 divisions of data are required: training, validation and test/holdout data (31,43,48).

There are 2 main approaches in the literature to address the stability and reliability of SPLS results. The first approach is based on stability selection, which involves subsampling the (training) data and fitting SPLS with given regularization parameters. After many repetitions of this procedure, the variables selected in all SPLS models (64) or in a proportion of the SPLS models (65) are kept as the relevant variables to describe the association. This procedure can be applied to selecting variables (43,66) and to guiding model selection (40,67); however, it is computationally expensive and depends on additional parameters (e.g., number of repetitions, subsample sizes) that might need to be further optimized (68). The second approach is useful only for model evaluation and involves resampling the (overall) data (e.g., via bootstrapping) to provide confidence intervals for the SPLS model. Thus, this procedure is a complement to permutation testing; permutation testing indicates whether the identified SPLS model is different from a model obtained by chance, while bootstrapping assesses the reliability of the SPLS model (69,70).

Next, we discuss the 2 significant brain–behavior relationships identified in our dataset. The first associative effect in the main SPLS results captured a relationship between age (with the highest weight) and alcohol use and gray matter volume in the cingulate and association (frontoparietotemporal) cortices. The supplementary analysis with SPLS did not find a similar effect after regressing out age from the data, whereas the KCCA analysis included age and a mixture of other factors relating to anxiety, interpersonal difficulties, and externalizing (for details, see Supplemental Results). These results demonstrate that including demographic variables such as age in the SPLS/KCCA model enables identifying variables that covary/interact with demographic variables. Furthermore, the interactive deflation procedure can be seen as an alternative strategy to remove effects (e.g., age) from the data. The frontoparietotemporal areas show the biggest loss of gray matter during adolescence (71, 72, 73); accordingly, 2 recent studies using the same community sample showed that myelination is a key factor in cortical shrinkage in these regions (74,75). These areas also relate to alcohol use; landmark studies have shown that their structural attributes (especially in frontal cortex) can predict drinking behavior later in adolescence (76,77).

The second associative effect captured a relationship between depression-related items and mainly limbic regions. The main SPLS results included mainly items related to suicidality in this effect. The supplementary analyses with SPLS and KCCA selected additional items, including key depression symptoms (e.g., low mood, anhedonia, loss of energy and concentration) and core depressive beliefs (e.g., worthlessness, hopelessness, guilt, low self-esteem). Interestingly, some classic biological symptoms of depression (e.g., sleep, appetite, psychomotor disturbances) do not feature in the selected items, which are concentrated in the cognitive and behavioral aspects of depression. The brain regions with highest weights (in KCCA) or selected variables (in SPLS) were similar in all three analyses comprising amygdala, hippocampus, and parahippocampal gyrus, putamen, vermis, and insula. Despite having only 33 subjects with depression in this sample, there is a remarkable degree of overlap between these areas and those associated with depression in much larger studies. A large meta-analysis of voxel-based morphometry studies (*n* = 4101 major depressive disorder [MDD] subjects) (78) also found gray matter differences in depression in insula, inferior frontal gyrus, hippocampal areas, caudate, and fusiform gyrus (and in vermis in bipolar disorder), all of which feature in this latent dimension. Whether volumes of other subcortical regions such as amygdala and putamen contribute to depression risk is more controversial; large univariate analyses have not found significant associations with depression (79,80).

Although the specificity of these findings for depression is unclear—similar hippocampal and subcortical volume associations are seen in posttraumatic stress disorder (81) and attention-deficit/hyperactivity disorder (82), respectively—even cross-disorder findings may be useful for predicting outcome and, in particular, treatment response. For example, in relatively small samples, insula volume has been shown to predict relapse in MDD (83), and a combination of amygdala, hippocampus, insula, and vermis (and 3 cortical areas) can predict treatment response to computerized cognitive behavioral therapy for MDD.

The identified latent dimensions also relate to existing RDoC domains, namely positive valence systems (i.e., reward anticipation and satiation that is excessive in the first [alcohol use] but impaired in the second [e.g., anhedonia]) and social processing (in KCCA only), in the attribution of negative and critical mental states to others. Another important element of the RDoC framework is the interaction of its domains with neurodevelopmental trajectories and environmental risk factors. Although our subjects were adolescents and young adults, our brain structure results were consilient with the adult depression literature (reviewed above). This is important because although some studies in children find hippocampal volume associations with both depression (84) and anxiety (85), a meta-analysis in adults with MDD concluded that hippocampal volume associations were absent at first episode (79). Indeed, our previous study of functional imaging data in this dataset revealed 2 latent dimensions of depression that had opposite relationships with age, one of which related to trauma (sexual abuse) (86).

This study has some limitations. The sample size is modest, especially for participants with depression (*n* = 33). This, combined with the likely heterogeneity of the disorder, makes the results for the depression-related modes somewhat unstable; that is, although the selected behavioral variables are similar, some of the out-of-sample correlations are close to zero. Validation of our SPLS/KCCA models in a larger dataset of healthy adolescents and young adults and adolescents and young adults with depression would further strengthen the generalizability of our findings. Furthermore, the inclusion of a broader selection of clinical disorders would reveal the specificity of these findings for depression rather than for psychological distress in general.

Finally, we suggest some key areas for future work. First, future studies should investigate other regularization strategies for CCA and PLS; for example, applying group sparsity can capture group structures in the data that might exist owing to either preprocessing (e.g., smoothing) or a biological mechanism (43). Second, nonlinear approaches [e.g., KCCA with nonlinear kernel (50)] could explore more complex relationships between brain and behavioral data. Third, regularized CCA and PLS approaches can be used to find associations across more than 2 types of data (56,87), which may enable a more complete description of latent neurobiological (and other) factors. Fourth, the obtained latent space could be embedded in a predictive model to enable predictions of future outcomes such as treatment response. Finally, further research should investigate how these latent dimensions relate to the currently used diagnostic categories.

In conclusion, we have shown that regularized multivariate methods, such as SPLS and KCCA, embedded in our novel framework yield stable results that generalize to holdout data. The identified multivariate brain–behavior relationships are in agreement with many established findings in the literature concerning age, alcohol use, and depression-related changes in brain volume. In particular, it is very encouraging that our depression-related results agree with a wider literature despite having only a small number of subjects with MDD. The depression-related dimension also contained largely cognitive and behavioral aspects of depression rather than its biological features. Altogether, we propose that SPLS/KCCA combined with our innovative framework provides a principled way to investigate basic dimensions of brain–behavior relationships and has great potential to contribute to a biologically grounded definition of psychiatric disorders.

## Supplementary Material

Supplementary Material

Key Resources Table
