# Supplementary material for: Multiple Holdouts With Stability: Improving the Generalizability of Machine Learning Analyses of Brain–Behavior Relationships
Source: Biol Psychiatry. 2020 Feb 15;87(4):368–76. doi: 10.1016/j.biopsych.2019.12.001 (PMC6970221; doi:10.1016/j.biopsych.2019.12.001)
Supplement: Supplementary Material [file mmc1.pdf]

Multiple Holdouts With Stability: Improving the Generalizability of Machine Learning Analyses of Brain-Behavior Relationships

Supplemental Information

Table of Contents

Supplemental Methods and Materials ... 2
Regularized CCA/PLS models ... 2
Canonical Correlation Analysis (CCA) ... 2
Partial Least Squares (PLS) ... 2
Regularized and Kernel CCA (RCCA/KCCA) ... 3
Sparse CCA and Sparse PLS (SCCA/SPLS) ... 4
Iterative solution for regularized CCA/PLS models ... 5
Projection and orthogonal projection ... 6
PLS-SVD deflation ... 7
CCA deflation ... 8
PLS mode A deflation ... 9
PLS1 and PLS2 deflation ... 10
Technical notes ... 10
Approximation of dual KCCA ... 10
Solving CCA and PLS through singular value decomposition ... 11
Model selection and statistical evaluation of regularized CCA/PLS ... 12
Multiple holdout framework with stability criterion ... 12
Generalizability-stability diagram ... 14
Data ... 16
Participants ... 16
Behavioral and demographic data ... 18
Structural MRI data ... 20
Brain weight visualization and summarization ... 21
Supplemental Results ... 23
Stability of the main SPLS analysis ... 23
SPLS analysis regressing out age ... 34
KCCA analysis ... 37
Supplemental References ... 46

Supplemental Methods and Materials

Regularized CCA/PLS models

In this section, we present the formulations of the CCA and PLS (1) models and their regularized versions considered in the paper.

Canonical Correlation Analysis (CCA)

CCA finds a pair of weights, $\boldsymbol{u}$ and $\boldsymbol{v}$, such that the correlation between the projections of the data matrices, $\boldsymbol{X}$ and $\boldsymbol{Y}$ onto these weights are maximised:

$$max_{\boldsymbol{u},\boldsymbol{v}}\ \boldsymbol{u}^T\boldsymbol{X}^T\boldsymbol{Y}\boldsymbol{v}$$

$$subject\ to\ \ ||\boldsymbol{X}\boldsymbol{u}||_2^2 = 1,$$

$$||\boldsymbol{Y}\boldsymbol{v}||_2^2 = 1 \qquad \text{(Eq. 1)}$$

where $\boldsymbol{X}$ and $\boldsymbol{Y}$ are data matrices containing one (standardized) brain or behavioral variable per column, respectively, and one example/sample per row, $||\boldsymbol{X}\boldsymbol{u}||_2^2$ and $||\boldsymbol{Y}\boldsymbol{v}||_2^2$ refer to the squared L2-norm constraint on the projections of the data ($\boldsymbol{X}\boldsymbol{u}$, $\boldsymbol{Y}\boldsymbol{v}$). We note that $||\cdot||_2$ denotes L2-norm of a vector and $\cdot^T$ denotes transpose of a vector/matrix. Notably, $\boldsymbol{x}^T\boldsymbol{x} = ||\boldsymbol{x}||_2^2$ for any vector x.

Partial Least Squares (PLS)

PLS finds a pair of weights, $\boldsymbol{u}$ and $\boldsymbol{v}$, such that the covariance between the projections of $\boldsymbol{X}$ and $\boldsymbol{Y}$ onto these weights are maximised. It can be written in a similar form to the CCA optimization problem with modified constraints:

$$max_{\boldsymbol{u},\boldsymbol{v}}\ \boldsymbol{u}^T\boldsymbol{X}^T\boldsymbol{Y}\boldsymbol{v}$$

$$subject\ to\ \ ||\boldsymbol{u}||_2^2 = 1,$$

$$||\boldsymbol{v}||_2^2 = 1 \qquad \text{(Eq. 2)}$$

where $||\boldsymbol{u}||_2^2$ and $||\boldsymbol{v}||_2^2$ are the squared L2-norm constraints on the weights ($\boldsymbol{u}$, $\boldsymbol{v}$).

Regularized and Kernel CCA (RCCA/KCCA)

To control the complexity of the CCA model and the risk of overfitting, a regularised version of CCA (RCCA) was proposed by Hardoon et al. (2) introducing regularization parameters of c_x and c_y, such that they impose squared L2-norm constraints both on the projections of the data ($\boldsymbol{Xu}$, $\boldsymbol{Yv}$) and the weights ($\boldsymbol{u}$, $\boldsymbol{v}$):

$$max_{\boldsymbol{u},\boldsymbol{v}}\ \boldsymbol{u}^T\boldsymbol{X}^T\boldsymbol{Y}\boldsymbol{v}$$

$$subject\ to\ \ (1-c_x)\|\boldsymbol{Xu}\|_2^2 + c_x\|\boldsymbol{u}\|_2^2 = 1,$$

$$(1-c_y)\|\boldsymbol{Yv}\|_2^2 + c_y\|\boldsymbol{v}\|_2^2 = 1 \qquad \text{(Eq. 3)}$$

The regularization parameters (c_x, c_y) vary from 0 to 1, i.e., in this formulation regularized CCA can be seen as a mixture of CCA and PLS with two cornerstone solutions $\{c_x, c_y\} = \{0, 0\}$ and $\{c_x, c_y\} = \{1, 1\}$ resulting in CCA and PLS, respectively. Therefore, PLS can be seen as a fully regularized CCA resulting in more stable solutions which are less likely to c

Kernel regularized CCA (KCCA) is an extension of the aforementioned regularised CCA, where the optimisation problem is solved by a kernel trick, when the above primal formulation (Eq. 3) is changed to a dual formulation (1):

$$max_{\boldsymbol{\alpha}_x\boldsymbol{\alpha}_y}\ \boldsymbol{\alpha}_x^T\boldsymbol{K}_x\boldsymbol{K}_y\boldsymbol{\alpha}_y$$

$$subject\ to\ \ (1-c_x)\boldsymbol{\alpha}_x^T\boldsymbol{K}_x^2\boldsymbol{\alpha}_x + c_x\boldsymbol{\alpha}_x^T\boldsymbol{K}_x\boldsymbol{\alpha}_x = 1,$$

$$(1-c_y)\boldsymbol{\alpha}_y^T\boldsymbol{K}_y^2\boldsymbol{\alpha}_y + c_y\boldsymbol{\alpha}_y^T\boldsymbol{K}_y\boldsymbol{\alpha}_y = 1 \qquad \text{(Eq. 4)}$$

where $\boldsymbol{\alpha}_x, \boldsymbol{\alpha}_y$ are the dual weights and $\boldsymbol{K}_x, \boldsymbol{K}_y$ are the kernels matrices for $\boldsymbol{X}, \boldsymbol{Y}$. Crucially, this optimization does not depend on the dimensionality of the features (as it is the case for RCCA) but the number of the examples, thus KCCA is computationally more efficient than RCCA when the number of features is much larger than the number of examples. In our implementation, we used linear kernels ($\boldsymbol{K}_x = \boldsymbol{X}\boldsymbol{X}^T$ and $\boldsymbol{K}_y = \boldsymbol{Y}\boldsymbol{Y}^T$)

where the primal weights ($\boldsymbol{u}$, $\boldsymbol{v}$) can be expressed as a linear function of the input data as follows:

$$\boldsymbol{u} = \boldsymbol{X}^T \boldsymbol{\alpha}_x$$
$$\boldsymbol{v} = \boldsymbol{Y}^T \boldsymbol{\alpha}_y \qquad \text{(Eq. 5)}$$

Importantly, $\boldsymbol{u}$ and $\boldsymbol{v}$ have the same dimensionality as the original brain and behavioral data, respectively, and show the contribution of the primal features (i.e., original brain and behavioral variables) to the associative effect facilitating the interpretation of the results.

For computational efficiency, we used the following approximation of the dual KCCA formulation in Eq. 4:

$$max_{\ \boldsymbol{w}_x \boldsymbol{w}_y} \boldsymbol{w}_x^T \boldsymbol{R}_x^T \boldsymbol{R}_y \boldsymbol{w}_y$$
$$subject\ to\ \ (1 - c_x)\|\boldsymbol{R}_x \boldsymbol{w}_x\|_2^2 + c_x\|\boldsymbol{w}_x\|_2^2 = 1,$$
$$(1 - c_y)\left\|\boldsymbol{R}_y \boldsymbol{w}_y\right\|_2^2 + c_y\left\|\boldsymbol{w}_y\right\|_2^2 = 1 \qquad \text{(Eq. 6)}$$

where $\boldsymbol{w}_x, \boldsymbol{w}_y$ are the weights of the approximation problem and $\boldsymbol{R}_x, \boldsymbol{R}_y$ are new matrices obtained by performing eigenvalue decomposition of the kernel metrices (for details, see section technical notes below). Finally, the primal weights ($\boldsymbol{u}$, $\boldsymbol{v}$) can be recovered as:

$$\boldsymbol{u} = \boldsymbol{X}^T \boldsymbol{R}_x (\boldsymbol{R}_x \boldsymbol{R}_x^T)^{-1} \boldsymbol{w}_x$$
$$\boldsymbol{v} = \boldsymbol{Y}^T \boldsymbol{R}_y (\boldsymbol{R}_y \boldsymbol{R}_y^T)^{-1} \boldsymbol{w}_y \qquad \text{(Eq. 7)}$$

to enable inference about the primal features (i.e., brain and behavioral variables).

Sparse CCA and Sparse PLS (SCCA/SPLS)

Witten et al. (3) proposed a sparse version of CCA (SCCA), which applies elastic net regularization combining both L1-norm (denoted by $\|\cdot\|_1$) and squared L2-norm constraints:

$$max_{\boldsymbol{u},\boldsymbol{v}}\ \boldsymbol{u}^T\boldsymbol{X}^T\boldsymbol{Y}\boldsymbol{v}$$

$$subject\ to\ \ \|\boldsymbol{X}\boldsymbol{u}\|_2^2 = 1, \|\boldsymbol{Y}\boldsymbol{v}\|_2^2 = 1,$$

$$\|\boldsymbol{u}\|_1 \leq c_u, \|\boldsymbol{v}\|_1 \leq c_v \qquad \text{(Eq. 8)}$$

Due to the high-dimensionality of the data, the variance matrices were substituted by identity matrices (denoted by $\mathbf{I}$, i.e., $\boldsymbol{X}^T\boldsymbol{X} = \boldsymbol{I}, \boldsymbol{Y}^T\boldsymbol{Y} = \boldsymbol{I}$) leading to the following optimisation problem (3):

$$max_{\boldsymbol{u},\boldsymbol{v}}\ \boldsymbol{u}^T\boldsymbol{X}^T\boldsymbol{Y}\boldsymbol{v}$$

$$subject\ to\ \ \|\boldsymbol{u}\|_2^2 = 1, \|\boldsymbol{v}\|_2^2 = 1,$$

$$\|\boldsymbol{u}\|_1 \leq c_u, \|\boldsymbol{v}\|_1 \leq c_v \qquad \text{(Eq. 9)}$$

which is essentially a sparse version of PLS (SPLS) (see Eq. 2). The elastic net regularization on the weight vectors enforces sparse weights due to the L1-norm constraint (i.e., it selects a subset of variables in each set of data) and enables simultaneously the selection of correlated variables due to the L2-norm constraint (4). This is one of the most popular SCCA/SPLS methods (5–13) and the one we used in our SPLS implementation.

Iterative solution for regularized CCA/PLS models

In this section, we describe the iterative solutions used for solving regularized CCA/PLS in the paper. Although there are standard methods (e.g. SVD, see section technical notes below) to estimate all weights or relationships/associative effects for CCA/PLS at once, it is desired to have an iterative solution for regularized CCA/PLS to be able to optimize the regularization parameters (e.g. sparsity level) for each associative effect independently. In such iterative solution, one pair of weights (e.g. $\boldsymbol{u}_i, \boldsymbol{v}_i$, where subscript i denotes the ith associative effect) is estimated at a time and then removed from the data (by a process known as deflation). The same process is repeated to find consecutive pairs of weights (14, 15). Furthermore, the iterative

solution allows choosing a specific deflation strategy (e.g. with desirable orthogonality properties) and is used to define different types of PLS variants.

CCA/PLS models and their regularized variants can be divided in two main groups based on their modelling aim. CCA/PLS can be used either for identifying associations between two sets of data (symmetric variants) or to predict one set of data from the other set (asymmetric variants) (14). Depending on the deflation strategy there are different types of symmetric (e.g. CCA, PLS mode A, PLS-SVD) and asymmetric (e.g. PLS1/PLS2, where PLS1 refers to the variant with a single output variable and PLS2 refers to the variant with multiple output variables) variants (14, 16, 17).

As deflation of a data matrix D can be seen as an orthogonal projection to the subspace of D (for details see below), the iterative deflation of D will provide orthogonal effects. Depending on the deflation strategy this will provide:

- orthogonal weight vectors ($\boldsymbol{u}, \boldsymbol{v}$) in case of PLS-SVD, e.g. $\boldsymbol{u}_i \perp \boldsymbol{u}_{i+1}$, $\boldsymbol{v}_i \perp \boldsymbol{v}_{i+1}$
- orthogonal latent variables for both data ($\boldsymbol{Xu}, \boldsymbol{Yv}$, i.e., projections of the data $\boldsymbol{X}$, $\boldsymbol{Y}$ onto the weights $\boldsymbol{u}$, $\boldsymbol{v}$) in case of CCA and PLS mode A, i.e., $\boldsymbol{X}_i\boldsymbol{u}_i \perp \boldsymbol{X}_{i+1}\boldsymbol{u}_{i+1}$, $\boldsymbol{Y}_i\boldsymbol{v}_i \perp \boldsymbol{Y}_{i+1}\boldsymbol{v}_{i+1}$.
- orthogonal latent variables only for one data ($\boldsymbol{Xu}$) in case of PLS1/PLS2, i.e., $\boldsymbol{X}_i\boldsymbol{u}_i \perp \boldsymbol{X}_{i+1}\boldsymbol{u}_{i+1}$

To better understand these different deflation strategies, we first introduce the mathematical concept of *projection* and *orthogonal projection,* and then describe the formulations for the different deflation strategies.

Projection and orthogonal projection

The projection of vector $\boldsymbol{x}$ onto vector $\boldsymbol{w}$ is given as:

$$P(\boldsymbol{x}) = \frac{\langle \boldsymbol{w}, \boldsymbol{x} \rangle}{||\boldsymbol{w}||_2^2}\boldsymbol{w} \quad \text{(Eq. 10)}$$

where $\langle \boldsymbol{w}, \boldsymbol{x} \rangle$ is the scalar product between $\boldsymbol{x}$ and $\boldsymbol{w}$ and $P(\boldsymbol{x})$ is the projection. Using the properties of a scalar product, the projection can be reformulated as:

$$P(\boldsymbol{x}) = \frac{\boldsymbol{w}\boldsymbol{w}^T\boldsymbol{x}}{||\boldsymbol{w}||_2^2} \quad \text{(Eq. 11)}$$

The orthogonal projection projects the data to the orthogonal complement of $P(\boldsymbol{x})$ (i.e., orthogonal to $P(\boldsymbol{x})$), which is given by:

$$P^{\perp}(\boldsymbol{x}) = \boldsymbol{x} - P(\boldsymbol{x}) \quad \text{(Eq. 12)}$$

Substituting Eq. 11, the orthogonal projection can be rewritten as:

$$P^{\perp}(\boldsymbol{x}) = \boldsymbol{x} - \frac{\boldsymbol{w}\boldsymbol{w}^T\boldsymbol{x}}{||\boldsymbol{w}||_2^2} = \left(\boldsymbol{I} - \frac{\boldsymbol{w}\boldsymbol{w}^T}{||\boldsymbol{w}||_2^2}\right)\boldsymbol{x} \quad \text{(Eq. 13)}$$

In the following, we will demonstrate that each deflation strategy is a subcase of this equation.

PLS-SVD deflation

In case of PLS-SVD, the orthogonal projection of the rows of the data matrices $\boldsymbol{X}, \boldsymbol{Y}$ are applied onto the weights $\boldsymbol{u}, \boldsymbol{v}$:

$$P^{\perp}(\boldsymbol{X}^T) = \left(\boldsymbol{I} - \frac{\boldsymbol{u}\boldsymbol{u}^T}{||\boldsymbol{u}||_2^2}\right)\boldsymbol{X}^T$$

$$P^{\perp}(\boldsymbol{Y}^T) = \left(\boldsymbol{I} - \frac{\boldsymbol{v}\boldsymbol{v}^T}{||\boldsymbol{v}||_2^2}\right)\boldsymbol{Y}^T$$

(Eq. 14)

Using row vectors of $\boldsymbol{X}, \boldsymbol{Y}$ instead of their column vectors and given the constraints of PLS ($||\boldsymbol{u}||_2^2 = 1$, $||\boldsymbol{v}||_2^2 = 1$, see Eq. 2), the orthogonal projections become:

$$P^{\perp}(\boldsymbol{X}) = \boldsymbol{X}(\boldsymbol{I} - \boldsymbol{u}\boldsymbol{u}^T)$$

$$P^{\perp}(\boldsymbol{Y}) = \boldsymbol{Y}(\boldsymbol{I} - \boldsymbol{v}\boldsymbol{v}^T) \quad \text{(Eq. 15)}$$

Therefore, the deflation of PLS-SVD can be written as:

$$\boldsymbol{X}_{i+1} \leftarrow \boldsymbol{X}_i(\boldsymbol{I} - \boldsymbol{u}_i\boldsymbol{u}_i{}^T)$$
$$\boldsymbol{Y}_{i+1} \leftarrow \boldsymbol{Y}_i(\boldsymbol{I} - \boldsymbol{v}_i\boldsymbol{v}_i{}^T) \qquad \text{(Eq. 16)}$$

where subscript i denotes the ith associative effect. As the subsequent weights $\boldsymbol{u}_{i+1}, \boldsymbol{v}_{i+1}$ can be expressed as the linear combination of rows of $\boldsymbol{X}_{i+1}, \boldsymbol{Y}_{i+1}$ and due to the definition of orthogonal projection (i.e., $\boldsymbol{X}_{i+1}, \boldsymbol{Y}_{i+1}$ are orthogonal to $\boldsymbol{u}_i, \boldsymbol{v}_i$, respectively, see Eqs. 12-13), this deflation will ensure that the subsequent weights will be orthogonal to each other, i.e., $\boldsymbol{u}_i \perp \boldsymbol{u}_{i+1}$, $\boldsymbol{v}_i \perp \boldsymbol{v}_{i+1}$. It also reflects that using this deflation strategy is equivalent to performing SVD decomposition at once (if no further constraint as e.g. regularization is applied).

CCA deflation

In case of CCA, the orthogonal projection of the columns of the data matrices $\boldsymbol{X}, \boldsymbol{Y}$ are applied onto the latent variables $\boldsymbol{Xu}, \boldsymbol{Yv}$:

$$P^{\perp}(\boldsymbol{X}) = \left(\boldsymbol{I} - \frac{\boldsymbol{Xu}(\boldsymbol{Xu})^T}{\|\boldsymbol{Xu}\|_2^2}\right)\boldsymbol{X}$$
$$P^{\perp}(\boldsymbol{Y}) = \left(\boldsymbol{I} - \frac{\boldsymbol{Yv}(\boldsymbol{Yv})^T}{\|\boldsymbol{Yv}\|_2^2}\right)\boldsymbol{Y} \qquad \text{(Eq. 17)}$$

which given the constraints of CCA ($\|\boldsymbol{Xu}\|_2^2 = 1$, $\|\boldsymbol{Yv}\|_2^2 = 1$, see Eq. 1) can be reformulated as:

$$P^{\perp}(\boldsymbol{X}) = \boldsymbol{X} - \boldsymbol{Xu}(\boldsymbol{Xu})^T\boldsymbol{X} = \boldsymbol{X}(\boldsymbol{I} - \boldsymbol{uu}^T\boldsymbol{X}^T\boldsymbol{X})$$
$$P^{\perp}(\boldsymbol{Y}) = \boldsymbol{Y} - \boldsymbol{Yv}(\boldsymbol{Yv})^T\boldsymbol{Y} = \boldsymbol{Y}(\boldsymbol{I} - \boldsymbol{vv}^T\boldsymbol{Y}^T\boldsymbol{Y}) \qquad \text{(Eq. 18)}$$

Therefore, the deflation of CCA can be written as:

$$\boldsymbol{X}_{i+1} \leftarrow \boldsymbol{X}_i\left(\boldsymbol{I} - \boldsymbol{u}_i\boldsymbol{u}_i{}^T\boldsymbol{X}_1{}^T\boldsymbol{X}_1\right)$$
$$\boldsymbol{Y}_{i+1} \leftarrow \boldsymbol{Y}_i\left(\boldsymbol{I} - \boldsymbol{v}_i\boldsymbol{v}_i{}^T\boldsymbol{Y}_1{}^T\boldsymbol{Y}_1\right) \qquad \text{(Eq. 19)}$$

Proofs on how to arrive to Eq. 19 (i.e., using $\boldsymbol{X}_1{}^T\boldsymbol{X}_1$, $\boldsymbol{Y}_1{}^T\boldsymbol{Y}_1$ instead of $\boldsymbol{X}_i{}^T\boldsymbol{X}_i$, $\boldsymbol{Y}_i{}^T\boldsymbol{Y}_i$) have been omitted for simplicity, please see (1) for further details.

Similarly, it can be shown that the deflation of KCCA (see Eq. 6) can be written as:

$$\boldsymbol{R}_{x_{i+1}} \leftarrow \boldsymbol{R}_{x_i}\left(\boldsymbol{I} - \boldsymbol{w}_{x_i}\boldsymbol{w}_{x_i}{}^T\left((1-c_{x_i})\boldsymbol{R}_{x_1}{}^T\boldsymbol{R}_{x_1} + c_{x_i}\boldsymbol{I}\right)\right)$$
$$\boldsymbol{R}_{y_{i+1}} \leftarrow \boldsymbol{R}_{y_i}\left(\boldsymbol{I} - \boldsymbol{w}_{y_i}\boldsymbol{w}_{y_i}{}^T\left((1-c_{y_i})\boldsymbol{R}_{y_1}{}^T\boldsymbol{R}_{y_1} + c_{y_i}\boldsymbol{I}\right)\right) \quad \text{(Eq. 20)}$$

PLS mode A deflation

In case of PLS mode A, similarly to CCA, the orthogonal projection of the columns of the data matrices $\boldsymbol{X}, \boldsymbol{Y}$ are applied onto the latent variables $\boldsymbol{Xu}, \boldsymbol{Yv}$ resulting in Eq. 17., which can be reformulated as:

$$P^{\perp}(\boldsymbol{X}) = \boldsymbol{X} - \frac{\boldsymbol{Xu}(\boldsymbol{Xu})^T\boldsymbol{X}}{\|\boldsymbol{Xu}\|_2^2} = \boldsymbol{X}\left(\boldsymbol{I} - \frac{\boldsymbol{uu}^T\boldsymbol{X}^T\boldsymbol{X}}{\|\boldsymbol{Xu}\|_2^2}\right)$$
$$P^{\perp}(\boldsymbol{Y}) = \boldsymbol{Y} - \frac{\boldsymbol{Yv}(\boldsymbol{Yv})^TY}{\|\boldsymbol{Yv}\|_2^2} = \boldsymbol{Y}\left(\boldsymbol{I} - \frac{\boldsymbol{vv}^T\boldsymbol{Y}^T\boldsymbol{Y}}{\|\boldsymbol{Yv}\|_2^2}\right) \quad \text{(Eq. 21)}$$

Defining loadings of $\boldsymbol{X}$ and $\boldsymbol{Y}$ as: $\boldsymbol{p}_i = \frac{\boldsymbol{X}_i{}^T\boldsymbol{X}_i\boldsymbol{u}_i}{\|\boldsymbol{X}_i\boldsymbol{u}_i\|_2^2}$, $\boldsymbol{q}_i = \frac{\boldsymbol{Y}_i{}^T\boldsymbol{Y}_i\boldsymbol{v}_i}{\|\boldsymbol{Y}_i\boldsymbol{v}_i\|_2^2}$, respectively, gives the final deflation form of PLS mode A:

$$\boldsymbol{X}_{i+1} \leftarrow \boldsymbol{X}_i(\boldsymbol{I} - \boldsymbol{u}_i\boldsymbol{p}_i{}^T)$$
$$\boldsymbol{Y}_{i+1} \leftarrow \boldsymbol{Y}_i(\boldsymbol{I} - \boldsymbol{v}_i\boldsymbol{q}_i{}^T) \quad \text{(Eq. 22)}$$

As the subsequent latent variables $\boldsymbol{X}_{i+1}\boldsymbol{u}_{i+1}, \boldsymbol{Y}_{i+1}\boldsymbol{v}_{i+1}$ can be expressed as the linear combination of columns of $\boldsymbol{X}_{i+1}, \boldsymbol{Y}_{i+1}$ and due to the definition of orthogonal projection (i.e., $\boldsymbol{X}_{i+1}, \boldsymbol{Y}_{i+1}$ are orthogonal to $\boldsymbol{X}_i\boldsymbol{u}_i$, $\boldsymbol{Y}_i\boldsymbol{v}_i$, respectively, see Eqs. 12-13), this deflation will ensure that the subsequent latent variables will be orthogonal to each other, i.e., $\boldsymbol{X}_i\boldsymbol{u}_i \perp \boldsymbol{X}_{i+1}\boldsymbol{u}_{i+1}$, $\boldsymbol{Y}_i\boldsymbol{v}_i \perp \boldsymbol{Y}_{i+1}\boldsymbol{v}_{i+1}$. Notably, if $\boldsymbol{X}^T\boldsymbol{X} = \boldsymbol{I}$ and $\boldsymbol{Y}^T\boldsymbol{Y} = \boldsymbol{I}$, it can be shown after simple transformations that $\boldsymbol{p}_i = \boldsymbol{u}_i$ and $\boldsymbol{q}_i = \boldsymbol{v}_i$, thus PLS mode A deflation will be equivalent to PLS-SVD deflation (Eq. 16).

PLS1 and PLS2 deflation

In case of PLS1 and PLS2, the same orthogonal projection is applied as in PLS mode A, however, as these traditional forms of PLS (15, 18) assume a prediction problem, i.e., predicting one set of data, $\boldsymbol{Y}$ (e.g. behavior) from another set, $\boldsymbol{X}$ (e.g. brain), only $\boldsymbol{X}$ needs to be deflated and $\boldsymbol{Y}$ can be left unchanged (1):

$$\boldsymbol{X}_{i+1} \leftarrow \boldsymbol{X}_i(\boldsymbol{I} - \boldsymbol{u}_i\boldsymbol{p}_i{}^T) \qquad \text{(Eq. 23)}$$

In our implementation, we chose PLS mode A deflation (Eq. 22) for SPLS as it is a symmetric variant finding brain-behavior associations where the directionality of brain vs. behavior or behavior vs. brain is not important. Furthermore, it provides orthogonal latent variables (i.e., projections of the data $\boldsymbol{X}, \boldsymbol{Y}$ onto the weights $\boldsymbol{u}$, $\boldsymbol{v}$) by considering the covariance of the variables in each set of data (i.e., $\boldsymbol{X}^T\boldsymbol{X}, \boldsymbol{Y}^T\boldsymbol{Y}$), which would be otherwise ignored by PLS-SVD. In case of KCCA, we used the deflation formulation described in Eq. 20.

Technical notes

Approximation of dual KCCA

At first, we calculate the eigenvalue decomposition of the kernel matrices, $\boldsymbol{K}_x$ and $\boldsymbol{K}_y$:

$$\boldsymbol{K}_x = \boldsymbol{V}_x\boldsymbol{L}_x\boldsymbol{V}_x^T$$

$$\boldsymbol{K}_y = \boldsymbol{V}_y\boldsymbol{L}_y\boldsymbol{V}_y^T \qquad \text{(Eq. 24)}$$

Then we use a lower-dimensional approximation of $\boldsymbol{K}_x, \boldsymbol{K}_y$, where the eigenvectors with the smallest eigenvalues are removed resulting in:

$$\boldsymbol{V}_x^* \sim \boldsymbol{V}_x$$

$$\boldsymbol{L}_x^* \sim \boldsymbol{L}_x$$

$$\boldsymbol{V}_y^* \sim \boldsymbol{V}_y$$

$$\boldsymbol{L}_y^* \sim \boldsymbol{L}_y \quad \text{(Eq. 25)}$$

Then new feature matrices are calculated as:

$$\boldsymbol{R}_x = \boldsymbol{V}_x^* \boldsymbol{L}_x^{*1/2}$$

$$\boldsymbol{R}_y = \boldsymbol{V}_y^* \boldsymbol{L}_y^{*1/2} \quad \text{(Eq. 26)}$$

Finally, we can view the dual formulation of KCCA (Eq. 4) as the primal formulation of RCCA (Eq. 3) with the feature vectors given by the columns of $\boldsymbol{R}_x$ and $\boldsymbol{R}_y$ (1).

Solving CCA and PLS through singular value decomposition

A popular implementation to solve CCA/PLS is using singular value decomposition (SVD):

$$\boldsymbol{M} = \boldsymbol{U\Sigma V}^T \quad \text{(Eq. 27)}$$

where $\boldsymbol{M} = (\boldsymbol{X}^T\boldsymbol{X})^{-1/2}\boldsymbol{X}^T\boldsymbol{Y}(\boldsymbol{Y}^T\boldsymbol{Y})^{-1/2}$ for CCA and $\boldsymbol{M} = \boldsymbol{X}^T\boldsymbol{Y}$ for PLS. The columns of $\boldsymbol{U}$ (i.e., $\boldsymbol{u}_1 \dots \boldsymbol{u}_n$, where the subscript indexes the columns referring to the associative effect) and $\boldsymbol{V}$ (i.e., $\boldsymbol{v}_1 \dots \boldsymbol{v}_n$, where the subscript indexes the columns referring to the associative effect) are singular vectors and correspond to the weights for PLS, however, the weights for CCA are computed with an additional transformation:

$$\boldsymbol{u} \leftarrow (\boldsymbol{X}^T\boldsymbol{X})^{-1/2}\boldsymbol{u}$$

$$\boldsymbol{v} \leftarrow (\boldsymbol{Y}^T\boldsymbol{Y})^{-1/2}\boldsymbol{v} \quad \text{(Eq. 28)}$$

As SVD results in orthonormal singular vectors, the weights of PLS are orthogonal to each other (i.e., $\boldsymbol{u}_i \perp \boldsymbol{u}_j, \boldsymbol{v}_i \perp \boldsymbol{v}_j$ for any $i \neq j$), whilst the weights for CCA are not orthogonal due to the additional operation in Eq. 28.

Model selection and statistical evaluation of regularized CCA/PLS

Multiple holdout framework with stability criterion

Our proposed framework performs model selection and model evaluation on multiple random subsets of the data. It is an extension of the multiple holdout framework of Monteiro et al. (12) combined with a stability criterion adapted from Baldassare et al. (19). In particular, 1) we used generalizability (measured as average out of sample correlation) and stability (measured as average overlap or correlation of the weights across the different data splits for SPLS and KCCA, respectively) as joint criterion for selecting regularization parameters in 50 random subsamples (Figure 2); 2) we performed a single hypothesis testing for the average holdout correlation across the different splits; 3) for each significant associative effect we presented results from the split that has the best combination of generalizability and stability.

In the following, we describe the current framework step by step (Figure 2, Supplemental Figure S1). First, we split (outer split) the brain data matrices ($\boldsymbol{X}$ for SPLS and $\boldsymbol{R}_x$ for KCCA, respectively) and behavioral data matrices ($\boldsymbol{Y}$ for SPLS and $\boldsymbol{R}_y$ for KCCA, respectively) randomly into optimization set (80% of the overall data) and holdout set (20% of the overall data). Then we further split (inner split) the optimization set 50 times into a training set (80% of the optimisation set) and a validation set (20% of the optimisation set). The inner split is used to optimize the regularization parameter and the outer split is used for significance testing. More specifically, for each combination of regularization parameters the SPLS/KCCA model is fitted on the training set, and the validation set is projected onto the resulting weight vectors. Across the 50 splits, this process yields, for each regularization parameter combination, average out-of-sample correlations on the validation set (measuring generalizability) and average corrected overlaps or correlation of the weights, for SPLS and KCCA,

respectively (measuring stability). The optimal combination of regularization parameters (c_u and c_v) are chosen based on a joint generalizability-stability criterion (explained below). These selected regularization parameters are then used to fit the SPLS/KCCA models on the overall optimisation set and the holdout set is projected onto the resulting weight vectors ($\boldsymbol{u}$ and $\boldsymbol{v}$) to compute holdout correlation. The above procedure is repeated 10 times in total, yielding 10 holdout correlations.

Finally, we perform a permutation test to assess the significance of the average holdout correlations across the 10 different splits. In the permutation test, we shuffle the order of the subjects (i.e., rows) in matrix $\boldsymbol{Y}$ to remove the associations between the brain and the behavioral data. We then fit the SPLS/KCCA models with the optimal regularization parameter (obtained from the original data) and project the holdout set onto the resulting weight vectors. Similarly to the original (i.e., unpermuted) data, permuted average holdout correlations are calculated across the 10 outer splits. Finally, we test the following null hypothesis: 'there is no reproducible relationship between the brain and behavioral data'. More specifically, we test if the average holdout correlation (measuring generalizability) obtained from the original data is not different from the one obtained with the permuted data given the optimized regularization parameters:

$$p = \frac{1}{B+1}\sum_{i=1}^{B+1}[\rho \geq \rho_b] \quad \text{(Eq. 29)}$$

where the so-called Iverson bracket $[\cdot]$ has the value 1 for a true condition and 0 for a false condition, and ρ and ρ_b are the average holdout correlation obtained from the original and permuted data, respectively ($B+1$ denotes that ρ is also added to the permutation distribution).

If the null hypothesis is rejected (i.e., we identify a significant brain-behavior association), the data matrices ($\boldsymbol{X}, \boldsymbol{Y}$ for SPLS, $\boldsymbol{R}_x, \boldsymbol{R}_y$ for KCCA) were deflated as

discussed in the previous section. For visualization purposes, we present the model with the best combination of generalizability and stability criterion across the different splits. Finally, all the above procedure is repeated to find additional brain-behavior associations.

Importantly, we apply a stratified splitting procedure both in the outer and the inner splits of the data to keep the proportion of depressed and healthy participants similar across data splits. In order to avoid any dependence between the training and validation sets during the regularization parameter optimization and between the optimization and holdout sets during the training of the final model with the selected regularization parameters, procedures for standardizing and deconfounding the data were estimated based on the training set and applied to the validation and holdout sets. The deflation was performed only on the training and the optimization sets. For estimating the second and subsequent brain-behavior associations, the data was not further standardized and deconfounded. Similarly, the permutation of the data was performed respecting the outer splits of the data, i.e., permuted within the optimization set and the holdout set separately. We used average correlation between the projections as a metric to measure generalizability instead of the absolute mean correlation, since the latter one was too conservative leading to a decrease in statistical power.

Generalizability-stability diagram

To understand the generalizability-stability diagram (Supplemental Figure S1), we introduce some measures taken from Baldassare et al. (19). The *support of a weight vector* is the set of its non-zero elements:

$$\boldsymbol{I}_i = \{k \mid w_k \neq 0\} \qquad \text{(Eq. 30)}$$

where $\{\cdot\}$ is a set, $\boldsymbol{I}_i$ is the support of the ith model, and $\boldsymbol{w}_k$ is the kth element of the weight vector. *Sparsity* is the relative number of the non-zero elements of a weight vector:

$$S = \frac{|\boldsymbol{I}|}{p} \quad \text{(Eq. 31)}$$

where $|\cdot|$ is the number of elements of a set, and p is the number of elements of the weight vector. We then define the *corrected pairwise overlap* of two weight vectors as:

$$O_{i,j} = \frac{|\boldsymbol{I}_i \cap \boldsymbol{I}_j| - E}{max(|\boldsymbol{I}_i|, |\boldsymbol{I}_j|)} \quad \text{(Eq. 32)}$$

where E is the expected overlap between the support of two random weight vectors with sparsity S_i and S_j, given the following formula:

$$E = p * S_i * S_j \quad \text{(Eq. 33)}$$

Finally, we define the similarity across weights as a measure of stability with:

- the *average corrected overlap* for SPLS:

$$\bar{O} = \frac{1}{N * (N-1)} \sum_{i \neq j = 1}^{N} O_{i,j} \quad \text{(Eq. 34)}$$

- the *average correlation* for KCCA:

$$\bar{C} = \frac{1}{N * (N-1)} \sum_{i \neq j = 1}^{N} C_{i,j} \quad \text{(Eq. 35)}$$

where $C_{i,j}$ is the absolute value of Pearson's correlation (to account for possible flipping due to the eigenvalue decomposition) between two weight vectors.

Our generalizability-stability diagram is an adaptation of the accuracy-stability diagram of Baldassarre et al. (19) to unsupervised models, which comprise overage out-of-sample correlations (measuring generalizability) and average similarity of the weights (measuring stability) (Supplemental Figure S1). The joint metric of generalizability and

stability is the Euclidean distance from the (1, 1) point in this diagram, which corresponds to perfect generalization and perfect stability of the model (the optimal regularization parameter is the one closest to the (1, 1) point in the diagram).

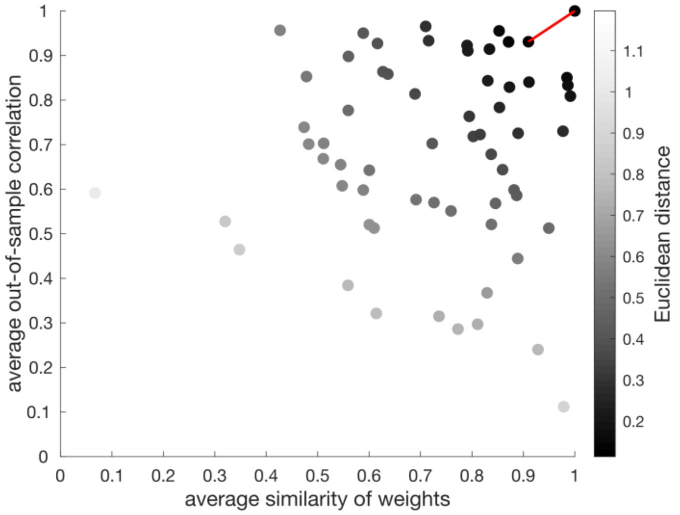

Supplemental Figure S1. Generalizability-stability diagram adapted from Baldassarre et al. (19) for selecting the regularization parameter in unsupervised models. The diagram illustrates average out-of-sample correlation (measuring generalizability on the validation sets) and average similarity of weights (measuring stability on the training sets). The joint metric of generalizability and stability is the Euclidean distance from the (1, 1) point in this diagram, which corresponds to perfect generalization and perfect stability of the model. The dots represent the different parameter combinations (c_u and c_v), and the red line indicates the smallest Euclidean distance pertaining to the best regularization parameter combination.

Data

Participants

The original sample was acquired by the University College London and University of Cambridge NeuroScience in Psychiatry Network (NSPN) research initiative, supported by a strategic award from the Wellcome Trust. It consisted of 2406 healthy participants

and 50 participants clinically diagnosed with depression (diagnosis and referral made by the participant's NHS care service) aged 14 to 24 years. The participants were recruited from schools, colleges, National Health Service (NHS) primary care and mental health services, and via direct advertisement in London and Cambridgeshire. A Magnetic Resonance Imaging (MRI) cohort was subsampled from the primary cohorts comprising a healthy cohort of 317 participants and a depression cohort of 37 participants. The data quality of the structural MR images were assessed by the Computational Anatomy Toolbox (CAT12; http://www.neuro.uni-jena.de/cat/) using the 'Check sample homogeneity' module and additional manual inspection, which resulted in the exclusion of 5 participants from the healthy cohort and 4 participants from the depression cohort. Therefore, the final healthy cohort consisted of 312 participants ($mean\ age = 19.14 \pm 2.93$, 156 females), and the final depression cohort consisted of 33 participants ($mean\ age = 16.50 \pm 1.23$, 23 females).

Written, informed consent was obtained for all participants over the age of 16 years. For participants under the age of 16, written informed assent was obtained for the participant and written informed consent from their parent/legal guardian. The study was ethically approved by the Cambridge Central Research Ethics Committee and conducted in accordance with NHS research governance standards.

All study data were collected and managed using REDCap electronic data capture tools hosted at the University of Cambridge (20). REDCap is a secure, web-based application designed to support data capture for research studies, providing 1) an intuitive interface for validated data entry; 2) audit trails for tracking data manipulation and export procedures; 3) automated export procedures for seamless data downloads to common statistical packages; and 4) procedures for importing data from external sources.

Behavioral and demographic data

Participants completed self-report questionnaires sent to their home and in the lab in assessment days as part of the NSPN data acquisition. We used a subset of questionnaires in the NSPN data that assess psychopathological symptoms, personality characteristics, mental wellbeing and IQ (21):

- Antisocial Behaviors Checklist (ABQ) (21) – self-report questionnaire for symptoms of antisocial behavior based on DSM-IV conduct disorder items. The questionnaire was designed solely for the purpose of the NSPN project (11 items).
- Antisocial Process Screening Device (APSD) (22) – self-report scale measuring psychopathic traits and antisocial behavior (20 items).
- Barratt Impulsive Scale (BIS) (23) – self-report questionnaire assessing personality and behavioral constructs of impulsiveness (30 items).
- Child and Adolescent Dispositions Scale (CADS) (24) – self-report measure of the three underlying dimensions of cognitive control of behavior: pro-sociability, negative emotionality and daring (57 items).
- Child Trauma Questionnaire (CTQ) (25) – self-report inventory screening for histories of abuse and neglect, which covers five types of maltreatment: emotional, physical, and sexual abuse, and emotional and physical neglect (28 items).
- Drugs Alcohol and Self-Injury (DASI) (21) – self-report measure assessing the frequency of drug and alcohol use as well as the frequency, methods and motives of non-suicidal self-harm acts. The questionnaire was designed solely for the purpose of the NSPN project (16 items).

- Inventory of Callous-Unemotional Traits (ICU) (26) – self-report inventory of assessing 3 domains of callous and unemotional traits: callousness, uncaring, and unemotional (24 items).
- Kessler Psychological Distress Scale (K10) (27) – self-report measurement of psychological distress (10 items).
- Leyton Obsessional Inventory (LOI) (28) – self-report questionnaire measuring obsessional and anxiety symptoms (11 items).
- Moods and Feelings Questionnaire (MFQ) (29) – self-report questionnaire measuring depressive symptoms in the last 2 weeks (33 items).
- Revised Children's Manifest Anxiety Scale (RCMAS) (30) – self-report questionnaire measuring anxiety symptoms (28 items).
- Rosenberg Self-Esteem Scale (SES) (31) – self-report questionnaire measuring global self-esteem or feelings of self-worth and self-acceptance (10 items).
- Schizotypal Personality Questionnaire (SPQ) (32) – self-report scale measuring schizotypal personality traits (74 items).
- Wechsler Abbreviated Scale of Intelligence (WASI) (33) – matrix reasoning and vocabulary subsets of the Wechsler Abbreviated Scale of Intelligence (WASI) designed to assess fluid and crystallized intelligence, respectively (2 items).
- Warwick Edinburgh Mental Wellbeing Scale (WEMWBS) (34) – self-report instruments spanning the theoretical distribution of common mental symptoms and wellbeing (14 items).

The socioeconomic status is a small-area model-based households in poverty estimate and reflects the proportion of poor households around the participant’s residence (see (https://www.ons.gov.uk/peoplepopulationandcommunity/personalandhouseholdfinan

ces/incomeandwealth/bulletins/smallareamodelbasedhouseholdsinpovertyestimatesenglandandwales/financialyearending2014 for full details). The index was calculated by a searchable web page provided by the Office of National Statistics, UK (https://www.ons.gov.uk/), however, we note that the online tool is no longer available, and only a downloadable updated dataset can be found here: https://www.ons.gov.uk/peoplepopulationandcommunity/personalandhouseholdfinances/incomeandwealth/datasets/householdsinpovertyestimatesformiddlelayersuperoutputareasinenglandandwales.

Structural MRI data

MRI data were acquired across the following three sites: 1) Medical Research Council Cognition and Brain Sciences Unit, Cambridge; 2) Wolfson Brain Imaging Centre, Cambridge; and 3) Wellcome Trust Centre for Neuroimaging, London. All sites were identically operating 3T whole-body MRI Systems (Magnetom TIM Trio, Siemens, Erlangen, Germany; VB17 software version) with standard 32-channel radio-frequency (RF) receive head coil and RF body coil for transmission. Between-site reliability and tolerability of all MRI procedures were satisfactorily assessed by a pilot study of five healthy volunteers at each site (35).

Structural brain scans were acquired using the quantitative Multi-Parameter-Mapping (MPM) protocol (35, 36) comprising three multi-echo 3D FLASH scans, one RF transmit field map and one static magnetic (B0) field map scan. Three different multi-echo FLASH scans were acquired with predominant T1-, PD-, and MT-weighting by appropriate choice of the repetition time (TR) and the flip angle α: TR/α = 18.7 ms/20° for the T1w scan and 23.7 ms/6° for the PDw and the MTw scans. Other acquisition parameters were 1 mm isotropic resolution, 176 sagittal partitions, field of view (FOV) = 256 × 240 mm, matrix size = 256 × 240 ×176 mm, parallel imaging using GRAPPA

factor 2 in phase-encoding (PE) direction, 6/8 partial Fourier in partition direction, non-selective RF excitation, read- out bandwidth BW = 425 Hz/pixel, RF spoiling phase increment = 50° with resulting total acquisition time of ~19 min.

Preprocessing of the structural MRI data was performed using SPM12 (www.fil.ion.ucl.ac.uk/spm) running on MATLAB (MathWorks). Individual differences of local grey matter volume was assessed using automatic segmentation of Magnetization Transfer Saturation (MT) parameter maps obtained from the MPM protocol (36). MT maps have been shown to improve contrast for segmentation of subcortical regions (37). The MT maps were segmented (38), normalised into MNI standard space using diffeomorphic registration and geodesic shooting (39), modulated by the determinant of the Jacobian of the deformation field, resampled to 2 x 2 x 2 mm^3, and spatially smoothed with a Gaussian kernel of 6 mm FWHM. Finally, these smoothed modulated grey matter volume probability maps were included in the SPLS/KCCA analyses.

Brain weight visualization and summarization

The brain weights were displayed separately for the subcortical structures (including hippocampus) and the cortex in Figure 2. The subcortical weights were overlaid on an MNI152 template from FSL (https://fsl.fmrib.ox.ac.uk/fsl/fslwiki/) (40) and visualized with nilearn (https://nilearn.github.io) (41). The cortical weights were visualized with BrainNet Viewer (http://www.nitrc.org/projects/bnv/) (42) using a default MNI152 glass brain of the software. For simplicity, the subcortical structures in Supplemental Figures S2 and S3 were projected on the medial wall of the cortical surface.

To guide interpretation, the brain weights were summarized by regions (Supplemental Tables S4, S6 and S8) using the Automated Anatomical Labeling atlas (43).

Specifically, we calculated the average weight for each region of the atlas and displayed the results for the top 20 regions ordered by absolute value.

Supplemental Results

Stability of the main SPLS analysis

To demonstrate the stability of the main SPLS results, here we present the results for the different data splits (Supplemental Tables S1-S3 and Supplemental Figures S2 and S3).

Supplemental Table S1. Summary of the main sparse partial least squares analysis for each data split. The data split marked in bold represents the best combination of generalizability (measured by the holdout correlation) and stability (measured by the average similarity of weights across the splits).

Data split	Holdout correlation	Brain stability[1]	Behavioral stability[2]	Brain sparsity[3]	Behavioral sparsity[4]
**First brain-behavior relationship**					
1	0.5946	0.3585	0.3262	28.03%	0.27%
2	0.5143	0.3592	0.3262	35.02%	0.27%
3	0.5501	0.3528	0.3262	35.13%	0.27%
4	0.2131	0.3492	0.5730	21.76%	1.65%
**5**	**0.5615**	**0.3465**	**0.5174**	**22.29%**	**2.20%**
6	0.3827	0.3155	0.2258	42.60%	5.49%
7	0.4310	0.2716	0.5519	50.60%	1.92%
8	0.4028	0.1561	0.5730	67.42%	1.65%
9	0.4984	0.3549	0.5730	22.45%	1.65%
10	0.5242	0.3256	0.5519	42.23%	1.92%
**Second brain-behavior relationship**					
1	0.1318	0.2262	0.4853	18.08%	1.37%
2	0.0995	0.1949	0.3753	24.25%	3.85%
3	0.1490	0.2167	0.3769	17.46%	3.57%
4	0.0394	0.1532	0.4853	30.37%	1.37%
5	0.1198	0.1689	0.3628	1.94%	3.57%
6	0.0032	0.2602	0.1544	7.97%	12.91%
**7**	**0.1236**	**0.3096**	**0.4416**	**4.58%**	**1.92%**
8	0.0240	0.2994	0.4853	4.42%	1.37%
9	0.1283	0.3024	0.2984	4.54%	3.30%
10	0.0229	0.2718	0.1164	4.85%	0.27%

[1-2] average corrected pairwise overlap of the brain/behavioral weights

[3-4] percentage of non-zero brain/behavioral weights

The first brain-behaviour relationship captured an association between age and drinking habits and a widespread set of frontoparietotemporal cortical regions (Figure 3). The results were rather robust across data splits (Supplemental Table S1). The holdout correlations were high and consistent between data splits (0.47 ± 0.04 SEM). The mean corrected overlap of the brain weights across the data splits was consistent and ranged between $\sim 0.3 - 0.4$ (0.32 ± 0.02 SEM). The mean corrected overlap of the behavioral weights ranged between $\sim 0.2 - 0.6$ (0.45 ± 0.04 SEM). The slightly lower values in 3 splits were due to only age being selected in those data splits. The number of selected behavioral variables were consistent across splits ($1.73\% \pm 0.48\%$ SEM). For the list of selected behavioral variables across splits see Supplemental Table S2. The number of selected brain variables were less sparse and ranged between $\sim 20 - 50\%$ ($36.75\% \pm 4.62\%$ SEM). The brain weights across splits are displayed in Supplemental Figure S2. The brain weights of the split that represents the best combination of generalizability (measured by the holdout correlation) and stability (measured by the average similarity of weights across the splits) is summarized in Supplemental Table S4.

Supplemental Table S2. Behavioral weights of the first brain-behavior relationship in the main sparse partial least squares analysis for each data split. APSD, Antisocial Process Screening Device; BIS, Barratt Impulsive Scale; CADS, Child and Adolescent Dispositions Scale; DASI, Drugs Alcohol and Self-Injury; ICU, Inventory of Callous-Unemotional Traits; SPQ, Schizotypal Personality Questionnaire.

Category	Behavioral variable	Weight
**Data split 1**		
Demographics	Age	1.0000
**Data split 2**		
Demographics	Age	1.0000
**Data split 3**		
Demographics	Age	1.0000

**Data split 4**		
Demographics	Age	0.8415
DASI	During the last month, how often did you drink spirits?	0.3156
DASI	In the last 6 months, how often have you been drunk in the way described in Q7?	0.2733
DASI	During the last month, how often did you drink beer or cider?	0.2411
DASI	Have you ever been drunk?	0.2408
DASI	During the last month, how often did you drink wine?	0.0393
**Data split 5**		
Demographics	Age	0.8760
DASI	During the last month, how often did you drink spirits?	0.2744
DASI	Have you ever been drunk?	0.2503
DASI	In the last 6 months, how often have you been drunk in the way described in Q7?	0.2427
DASI	During the last month, how often did you drink beer or cider?	0.1308
DASI	During the last month, how often did you drink wine?	0.1306
CADS	Are you more interested in sex than other people your age?	0.0396
CADS	Would you think it would be fun to watch two dogs fight?	0.0071
**Data split 6**		
Demographics	Age	0.6718
DASI	During the last month, how often did you drink spirits?	0.4653
DASI	In the last 6 months, how often have you been drunk in the way described in Q7?	0.3071
DASI	Have you ever been drunk?	0.2589
DASI	During the last month, how often did you drink beer or cider?	0.2553
DASI	During the last month, how often did you drink wine?	0.2000
CADS	Would you think it would be fun to watch two dogs fight?	0.1326
BIS	I change residences.	0.1306
CADS	Are you more interested in sex than other people your age?	0.0970
APSD	You tease or make fun of other people.	0.0775
Demographics	Poverty index	0.0729
ICU	I seem very cold and uncaring to others.	0.0300
Demographics	Male	0.0210
APSD	You do risky or dangerous things.	0.0093
SPQ	I get anxious when meeting people for the first time.	-0.0003
SPQ	I feel very uneasy talking to people I do not know well.	-0.0146
CADS	Do you like things to stay the same and not change?	-0.0189
Demographics	Female	-0.0210
ICU	I try not to hurt others' feelings.	-0.0400
CADS	Would you feel guilty if you did something that broke the law?	-0.0790
**Data split 7**		
Demographics	Age	0.8260
DASI	During the last month, how often did you drink spirits?	0.4123

DASI	In the last 6 months, how often have you been drunk in the way described in Q7?	0.3012
DASI	During the last month, how often did you drink beer or cider?	0.1677
DASI	Have you ever been drunk?	0.1469
DASI	During the last month, how often did you drink wine?	0.0847
CADS	Are you more interested in sex than other people your age?	0.0127
**Data split 8**		
Demographics	Age	0.8292
DASI	During the last month, how often did you drink spirits?	0.3880
DASI	In the last 6 months, how often have you been drunk in the way described in Q7?	0.3051
DASI	Have you ever been drunk?	0.2030
DASI	During the last month, how often did you drink beer or cider?	0.1449
DASI	During the last month, how often did you drink wine?	0.0814
**Data split 9**		
Demographics	Age	0.8578
DASI	During the last month, how often did you drink spirits?	0.3463
DASI	In the last 6 months, how often have you been drunk in the way described in Q7?	0.2412
DASI	Have you ever been drunk?	0.1893
DASI	During the last month, how often did you drink beer or cider?	0.1643
DASI	During the last month, how often did you drink wine?	0.1527
**Data split 10**		
Demographics	Age	0.8704
DASI	During the last month, how often did you drink spirits?	0.3346
DASI	In the last 6 months, how often have you been drunk in the way described in Q7?	0.2260
DASI	During the last month, how often did you drink beer or cider?	0.1895
DASI	Have you ever been drunk?	0.1778
DASI	During the last month, how often did you drink wine?	0.0828
CADS	Would you think it would be fun to watch two dogs fight?	0.0704

The second brain-behavior relationship captured an association between behavioral items related to depression, self-harm and grey matter volume in a small set of regions including the hippocampus, parahippocampal gyrus, insula, amygdala, pallidum and putamen (Figure 3). The results were slightly less robust across data splits (Supplemental Table S1). The holdout correlations were low and consistent between data splits (0.08 ± 0.02 SEM). The mean corrected overlap of the brain weights across splits was modest but consistent, ranged between $\sim 0.2 - 0.3$ (0.24 ± 0.02 SEM).

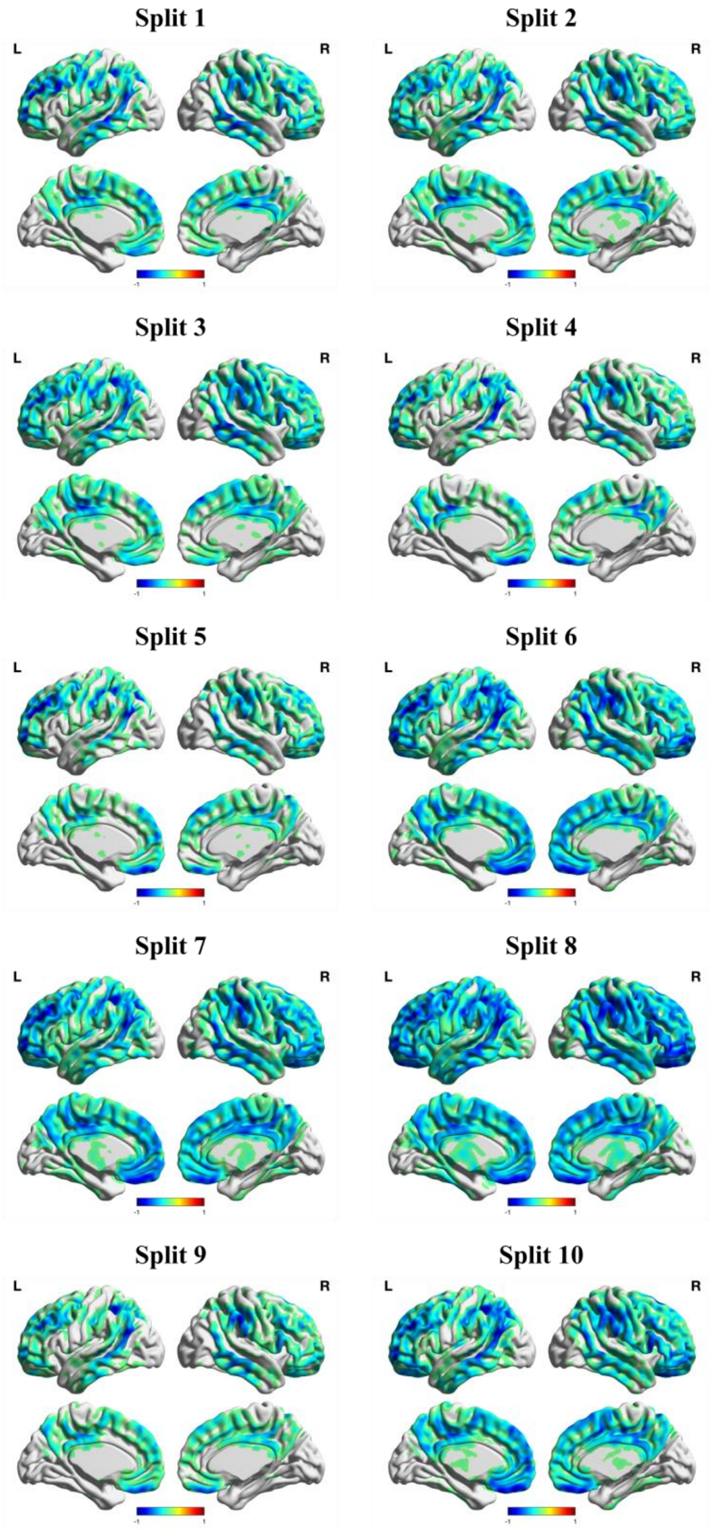

Supplemental Figure S2. Brain weights of the first brain-behavior relationship in the main sparse partial least squares analysis for each data split. The brain voxels are colour coded by weight, normalized for visualization purposes and displayed on MNI152 template for all cortical and subcortical regions.

The mean corrected overlap of the behavioral weights ranged between $\sim 0.2 - 0.5$ (0.36 ± 0.04 SEM). The number of selected behavioral variables were consistent across splits ($3.35\% \pm 1.13\%$ SEM). For the list of selected behavioral variables across splits see Supplemental Table S3. The number of selected brain variables were also rather sparse and ranged between $\sim 1 - 30\%$ ($11.85\% \pm 3.15\%$ SEM). The brain weights across splits are displayed in Supplemental Figure S3. The brain weights of the split that represents the best combination of generalizability (measured by the holdout correlation) and stability (measured by the average similarity of weights across the splits) is summarized in Supplemental Table S4.

Supplemental Table S3. Behavioral weights of the second brain-behavior relationship in the main sparse partial least squares analysis for each data split. Only up to the top 20 positive and top 20 negative weights are displayed. CADS, Child and Adolescent Dispositions Scale; DASI, Drugs Alcohol and Self-Injury; K10, Kessler Psychological Distress Scale; MFQ, Moods and Feelings Questionnaire; RCMAS, Revised Children's Manifest Anxiety Scale; SES, Rosenberg Self-Esteem Scale; SPQ, Schizotypal Personality Questionnaire; WEMWBS, Warwick Edinburgh Mental Wellbeing Scale.

Category	Behavioral variable	Weight
**Data split 1**		
MFQ	I thought about killing myself	0.6886
MFQ	I thought about dying	0.5857
MFQ	I thought that life was not worth living	0.3447
MFQ	I hated myself	0.2322
K10	During the last 30 days, about how often did you feel worthless?	0.1003
**Data split 2**		
MFQ	I thought about killing myself	0.5792
MFQ	I thought about dying	0.4547
MFQ	I thought that life was not worth living	0.4401
MFQ	I hated myself	0.3190
K10	During the last 30 days, about how often did you feel worthless?	0.2296
MFQ	I thought there was nothing good for me in the future	0.2044
K10	During the last 30 days, about how often did you feel depressed?	0.1397
RCMAS	I felt alone even when there were people with me	0.1395
SES	At times, I thought I was no good at all	0.1001
MFQ	I felt I was no good any more	0.0729
SES	I certainly felt useless at times	0.0675
MFQ	I didn't enjoy anything	0.0667

K10	During the last 30 days, about how often did you feel hopeless?	0.0603
MFQ	I thought my family would be better off without me	0.0293
**Data split 3**		
MFQ	I thought that life was not worth living	0.4791
MFQ	I thought about killing myself	0.4664
K10	During the last 30 days, about how often did you feel worthless?	0.4215
MFQ	I hated myself	0.3878
MFQ	I thought about dying	0.3572
K10	During the last 30 days, about how often did you feel hopeless?	0.1738
MFQ	I thought there was nothing good for me in the future	0.1332
SES	At times, I thought I was no good at all	0.1214
MFQ	I felt I was no good any more	0.1189
SES	I certainly felt useless at times	0.0987
K10	During the last 30 days, about how often did you feel depressed?	0.0981
RCMAS	I felt alone even when there were people with me	0.0254
K10	During the last 30 days, about how often did you feel so sad that nothing could cheer you up?	0.0215
**Data split 4**		
MFQ	I thought about killing myself	0.6958
MFQ	I thought about dying	0.5030
MFQ	I thought that life was not worth living	0.4726
K10	During the last 30 days, about how often did you feel worthless?	0.1530
MFQ	I hated myself	0.1272
**Data split 5**		
MFQ	I thought about killing myself	0.5481
MFQ	I hated myself	0.4542
RCMAS	Often I felt sick to my stomach	0.4261
MFQ	I thought about dying	0.3572
MFQ	I thought that life was not worth living	0.2609
DASI	Excluding the last month, why have you tried to hurt yourself without trying to kill yourself in the last 12 months?	0.2170
DASI	In the last month, how have you tried to hurt yourself without trying to kill yourself?	0.1402
MFQ	I was a bad person	0.1154
K10	During the last 30 days, about how often did you feel worthless?	0.1092
MFQ	I didn't enjoy anything	0.0920
RCMAS	I felt alone even when there were people with me	0.0140
CADS	Are you energetic when you have a job to do?	-0.0594
CADS	Are you cheerful?	-0.1094
**Data split 6**		
MFQ	I hated myself	0.3393
MFQ	I thought about killing myself	0.3250

MFQ	I thought that life was not worth living	0.2691
MFQ	I thought about dying	0.2682
DASI	Excluding the last month, why have you tried to hurt yourself without trying to kill yourself in the last 12 months?	0.2527
K10	During the last 30 days, about how often did you feel worthless?	0.2340
K10	During the last 30 days, about how often did you feel depressed?	0.1909
DASI	Excluding the last month, how have you tried to hurt yourself without trying to kill yourself in the last 12 months?	0.1903
K10	During the last 30 days, about how often did you feel hopeless?	0.1762
SES	I certainly felt useless at times	0.1717
DASI	In the last month, how have you tried to hurt yourself without trying to kill yourself?	0.1562
DASI	Excluding the last month, have you tried to hurt yourself on purpose without trying to kill yourself in the last 12 months?	0.1486
RCMAS	I felt alone even when there were people with me	0.1485
SES	At times, I thought I was no good at all	0.1461
MFQ	I did everything wrong	0.1443
MFQ	I didn't enjoy anything	0.1295
K10	During the last 30 days, about how often did you feel that everything was an effort?	0.1275
MFQ	I thought there was nothing good for me in the future	0.1046
SPQ	When you see other people talking to each other, do you often wonder if they are talking about you?	0.1023
MFQ	I felt I was no good any more	0.1019
SES	I was satisfied with myself	-0.0068
CADS	Are you proud of yourself?	-0.0189
CADS	Are you energetic when you have a job to do?	-0.0728
CADS	When you have something to do, are you determined to get it done?	-0.0780
SES	I felt that I was as good as anyone else	-0.1106
CADS	Are you cheerful?	-0.1196
WEMWBS	I've been feeling cheerful	-0.1303
SES	I was able to do things as well as most people	-0.1376
CADS	Do you feel confident that you can handle life's challenges?	-0.1543
CADS	Are you enthusiastic about life?	-0.1940
**Data split 7**		
MFQ	I thought about killing myself	0.7075
MFQ	I thought about dying	0.5250
MFQ	I thought that life was not worth living	0.4147
MFQ	I hated myself	0.2186
K10	During the last 30 days, about how often did you feel worthless?	0.0583
MFQ	I thought my family would be better off without me	0.0263

K10	During the last 30 days, about how often did you feel depressed?	0.0011
**Data split 8**		
MFQ	I thought about killing myself	0.6710
MFQ	I thought about dying	0.5326
MFQ	I thought that life was not worth living	0.4640
MFQ	I hated myself	0.2145
K10	During the last 30 days, about how often did you feel worthless?	0.0694
**Data split 9**		
DASI	Excluding the last month, why have you tried to hurt yourself without trying to kill yourself in the last 12 months?	0.5176
MFQ	I thought about killing myself	0.4650
MFQ	I thought about dying	0.4299
MFQ	I hated myself	0.3976
MFQ	I thought that life was not worth living	0.2263
DASI	Excluding the last month, how have you tried to hurt yourself without trying to kill yourself in the last 12 months?	0.2045
DASI	In the last month, why have you tried to hurt yourself without trying to kill yourself?	0.1568
DASI	Excluding the last month, have you tried to hurt yourself on purpose without trying to kill yourself in the last 12 months?	0.1476
RCMAS	Often I felt sick to my stomach	0.1239
MFQ	I was a bad person	0.0838
DASI	In the last month, have you tried to hurt yourself on purpose without trying to kill yourself?	0.0754
DASI	In the last month, how have you tried to hurt yourself without trying to kill yourself?	0.0747
**Data split 10**		
MFQ	I hated myself	1.0000

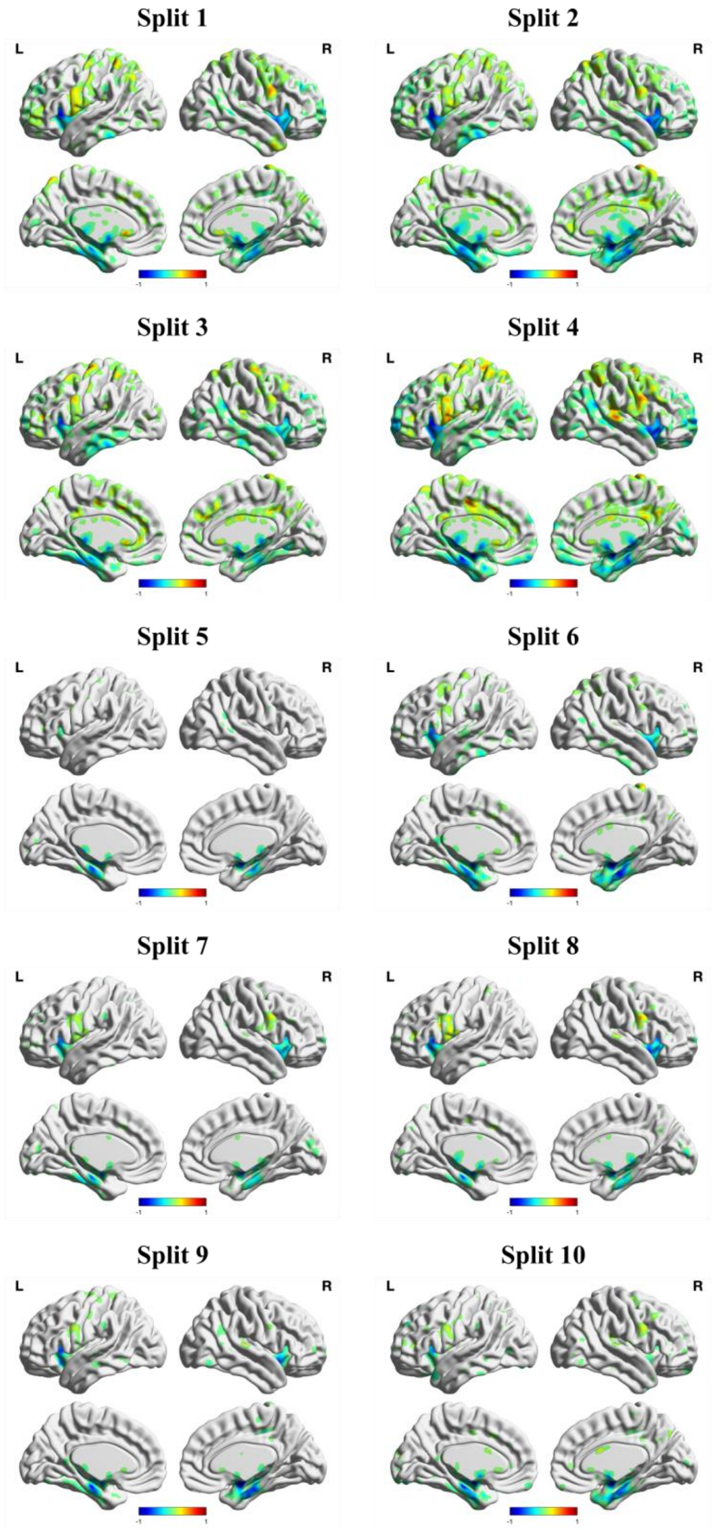

Supplemental Figure S3. Brain weights of the second brain-behavior relationship in the main sparse partial least squares analysis for each data split. The brain voxels are colour coded by weight, normalized for visualization purposes and displayed on MNI152 template for all cortical and subcortical regions.

Supplemental Table S4. Summary of the brain weights in the main sparse partial least squares analysis for the data split that represents the best combination of generalizability (measured by the holdout correlation) and stability (measured by the average brain and behavior stability). The brain weights are summarized by the top 20 brain regions using the Automated Anatomical Labeling atlas (43). L, Left; R, Right

Brain regions	Average weight	Number of voxels
**First brain-behavior relationship**		
L gyrus rectus	-0.0040	610
L middle cingulate & paracingulate gyri	-0.0029	1268
L inferior parietal gyrus (excluding supramarginal and angular gyri)	-0.0028	1368
R middle cingulate & paracingulate gyri	-0.0027	1401
L middle frontal gyrus	-0.0027	2377
L superior frontal gyrus (dorsolateral part)	-0.0024	2244
L angular gyrus	-0.0024	546
R supramarginal gyrus	-0.0024	1171
R medial orbital gyrus	-0.0024	424
R gyrus rectus	-0.0023	450
R superior frontal gyrus (medial part)	-0.0022	1113
L superior frontal gyrus (medial part)	-0.0020	1625
R inferior frontal gyrus (opercular part)	-0.0020	615
L postcentral gyrus	-0.0019	1925
L lateral orbital gyrus	-0.0018	120
L precentral gyrus	-0.0018	1415
L superior frontal gyrus (medial orbital part)	-0.0018	417
L supramarginal gyrus	-0.0017	546
R postcentral gyrus	-0.0016	1661
L superior parietal gyrus	-0.0016	905
**Second brain-behavior relationship**		
R amygdala	-0.0084	140
L hippocampus	-0.0076	589
L insula	-0.0054	738
R hippocampus	-0.0044	383
L amygdala	-0.0040	86
R insula	-0.0032	590
Vermis (part 3)	-0.0032	73
Vermis (parts 1-2)	-0.0028	16
R parahippocampal gyrus	-0.0027	359
L parahippocampal gyrus	-0.0017	200
R putamen	-0.0017	350
L cerebellum (part 3)	-0.0012	19
L fusiform gyrus	-0.0009	312
R caudate nucleus	-0.0008	143
R cerebellum (part 3)	-0.0008	22
L precentral gyrus	0.0007	315
R pallidum	-0.0005	46

R precentral gyrus	0.0004	265
L inferior frontal gyrus (triangular part)	-0.0004	229
R inferior frontal gyrus (orbital part)	-0.0004	42

SPLS analysis regressing out age

To discount the sampling bias of the subjects with depression who were younger on average ($mean\ age = 16.50 \pm 1.23$ SD in the depression cohort vs. $mean\ age = 19.14 \pm 2.93$ SD in the healthy cohort), we repeated the SPLS analysis, removing age from the behavioral/demographic variables and instead adding it to the confounds. Here, we identified one significant brain-behavior relationship ($p = .047$, Supplemental Figures S4 and S5, Supplemental Tables S5 and S6), which was very similar to the second depression-related effect of the main SPLS analysis (Figures 3 and 4B). Similar to the main text, we present the results for the split that represents the best combination of generalizability (measured by the holdout correlation) and stability (measured by the average similarity of weights across the splits).

Supplemental Table S5. Summary of the sparse partial least squares analysis regressing out age for each data split. The data split marked in bold represents the best combination of generalizability (measured by the holdout correlation) and stability (measured by the average similarity of weights across the splits).

Data split	Holdout correlation	Brain stability[1]	Behavioral stability[2]	Brain sparsity[3]	Behavioral sparsity[4]
**First brain-behavior relationship**					
1	0.1100	0.0647	0.2075	0.53%	14.33%
**2**	**0.1310**	**0.3206**	**0.2517**	**7.96%**	**22.87%**
3	0.1990	0.2060	0.2039	17.17%	29.48%
4	0.0746	0.2929	0.2549	12.16%	23.14%
5	0.0329	0.2968	0.3460	7.58%	0.28%
6	-0.0035	0.3312	0.2463	7.61%	18.46%
7	0.1222	0.1678	0.1119	2.09%	3.86%
8	-0.0438	0.2322	0.3460	17.19%	0.28%
9	0.0288	0.3295	0.3460	7.79%	0.28%
10	0.0056	0.3361	0.3460	8.01%	0.28%

[1-2] average corrected pairwise overlap of the brain/behavioral weights

[3-4] percentage of non-zero brain/behavioral weights

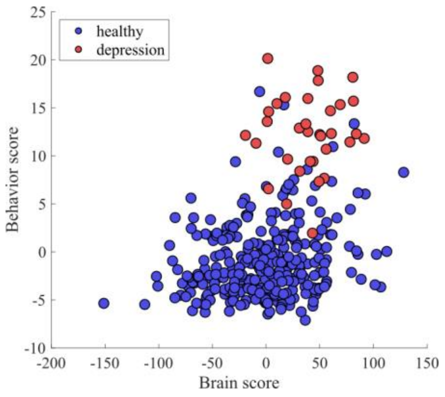

Supplemental Figure S4. Brain-behavior latent space identified by sparse partial least squares regressing out age. Scatterplot of the brain and behavioral scores with subjects colour coded by clinical diagnosis.

Indeed, all seven of the items (relating to suicidality and depressive cognitions) in the second brain-behavior relationship in the main SPLS analysis reappeared in this effect. The other items in this associative effect also related to suicidality, depressive cognitions (e.g. “I thought I was no good at all”, “I did everything wrong”, not being satisfied or proud with oneself), anhedonia (“I didn’t enjoy anything”, not enjoying being with other people) and loss of energy and motivation. The brain weights of this associative effect are similar to the main SPLS analysis and selected a very small set of brain regions including the hippocampus, parahippocampal gyrus, insula, amygdala, pallidum and putamen. The brain weights are summarized in Supplemental Table S6.

To quantitatively assess the similarity of the results, we calculated Pearson’s correlation between the brain and behavioral weights/scores of this analysis and the main SPLS analysis. To account for the sparsity of the weights, we used only the corresponding weight vectors of the two methods restricting them to the same length as the sparser solution. The similarity between the brain and the behavioral weights of the two methods are $r_{brain} = 0.6598$ and $r_{behavior} = 0.1856$, respectively; the similarity

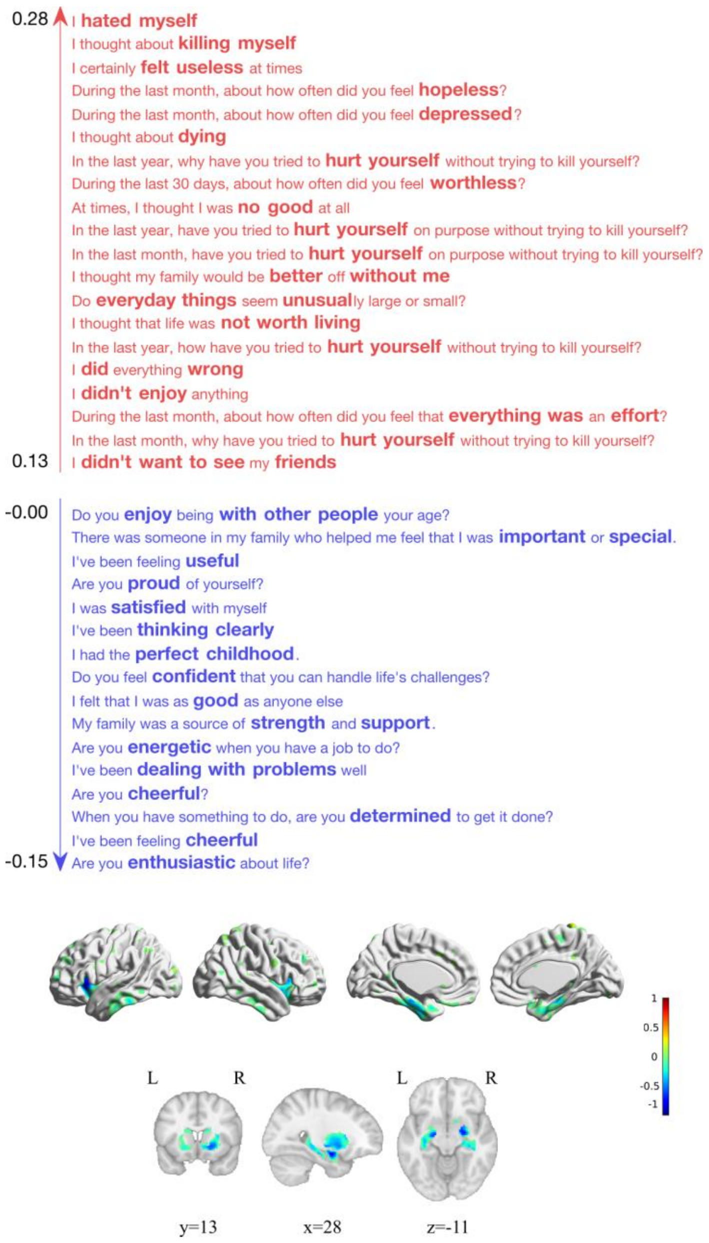

Supplemental Figure S5. Brain and behavioral weights of the sparse partial least squares analysis regressing out age. Only up to the top 20 positive and top 20 negative behavioral weights are displayed. The brain voxels are colour coded by weight, normalized for visualization purposes and displayed on MNI152 template for all cortical and subcortical regions.

between the brain and the behavioral scores of the two methods are $r_{brain} = 0.8711$ and $r_{behavior} = 0.8849$, respectively. We note that the low value for the similarity of the behavioral weights is probably because of the main SPLS analysis identified a very

sparse solution (including 7 variables in the model) whilst this analysis is less sparse (including 83 variables in the model).

Supplemental Table S6. Summary of the brain weights in the sparse partial least squares analysis regressing out age for the data split that represents the best combination of generalizability (measured by the holdout correlation) and stability (measured by the average similarity of weights across the splits). The brain weights are summarized by the top 20 brain regions using the Automated Anatomical Labeling atlas (43). L, Left; R, Right

**Brain regions**	**Average weight**	**Number of voxels**
R amygdala	-0.0106	215
R putamen	-0.0078	899
R pallidum	-0.0070	222
L hippocampus	-0.0065	684
L insula	-0.0058	962
R hippocampus	-0.0050	626
L amygdala	-0.0040	116
L parahippocampal gyrus	-0.0034	466
R parahippocampal gyrus	-0.0031	542
R insula	-0.0028	669
L putamen	-0.0018	587
L inferior frontal gyrus (triangular part)	-0.0013	392
L fusiform gyrus	-0.0010	461
R caudate nucleus	-0.0010	194
R thalamus	-0.0010	317
L caudate nucleus	-0.0008	216
L inferior temporal gyrus	-0.0008	536
R posterior orbital gyrus	-0.0007	128
R cerebellum (part 3)	-0.0006	16
R inferior frontal gyrus (triangular part)	-0.0006	165

KCCA analysis

To investigate how the proposed framework works with an alternative regularized approach, we used KCCA on the data from the main SPLS analysis including age as a behavioral/demographic variable. Here, we identified 2 significant brain-behaviour relationships (first associative effect: $p = .001$, Supplemental Figures S6A and S7, Supplemental Tables S7 and S8; second associative effect: $p = .006$, Supplemental

Figures S6B and S8, Supplemental Tables S7 and S8), which resembled to those identified by the main SPLS analysis (Figures 3-4A). Similar to the main analysis, we present the results for the split that represents the best combination of generalizability (measured by the holdout correlation) and stability (measured by the average similarity of weights across the splits).

Supplemental Table S7. Summary of the kernel canonical correlation analysis for each data split. The data split marked in bold represents the best combination of generalizability (measured by the holdout correlation) and stability (measured by the average similarity of weights across the splits).

Data split	Holdout correlation	Brain stability[1]	Behavioral stability[2]	Brain regularization[3]	Behavioral regularization[4]
**First brain-behavior relationship**					
1	0.0961	0.9945	0.1026	100%	95.26%
2	0.1483	0.9942	0.1040	100%	95.26%
3	0.1180	0.9947	0.0429	100%	95.26%
4	0.0893	0.9939	0.1012	100%	95.26%
5	0.1635	0.9940	0.0519	100%	95.26%
6	0.0728	0.9947	0.0912	100%	95.26%
7	0.1972	0.9938	0.0765	100%	95.26%
**8**	**0.2326**	**0.9948**	**0.0855**	**100%**	**95.26%**
9	-0.0452	0.9938	0.0862	100%	95.26%
10	0.2312	0.9938	0.0769	100%	95.26%
**Second brain-behavior relationship**					
1	0.2007	0.1311	0.9727	100%	100%
2	-0.0341	0.1412	0.9694	100%	100%
3	0.0443	0.1199	0.9732	100%	100%
4	-0.1255	0.1160	0.9681	100%	100%
5	0.2314	0.0558	0.9848	95.26%	100%
6	0.0278	0.1796	0.9709	100%	100%
**7**	**0.2488**	**0.1303**	**0.9725**	**100%**	**100%**
8	0.0884	0.1606	0.9735	100%	100%
9	0.2432	0.0911	0.9742	100%	100%
10	0.0455	0.1738	0.9741	100%	100%

[1-2] average pairwise correlation of the brain/behavioral weights

[3-4] value of regularization parameter in percentage

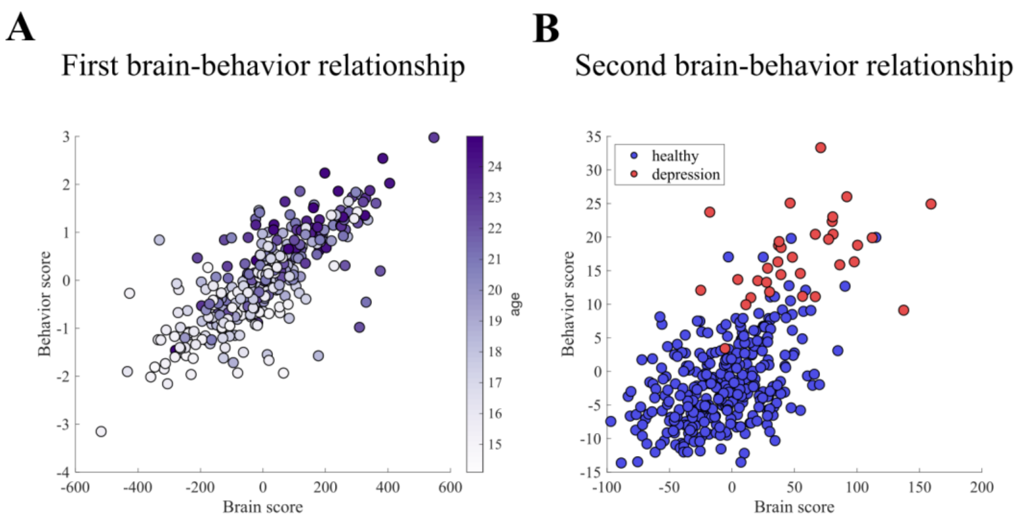

Supplemental Figure S6. Brain-behavior latent spaces identified by kernel canonical correlation analysis. The results are displayed for the data split that represents the best combination of generalizability (measured by the holdout correlation) and stability (measured by the average similarity of weights across the splits). **(A)** Scatterplot of the brain and behavioral scores of the first brain-behavior relationship with subjects colour coded by age. **(B)** Scatterplot of the brain and behavioral scores of the second brain-behavior relationship with subjects colour coded by clinical diagnosis.

The behavioral weights of the first brain-behavior relationship identified by KCCA analysis are, like the first effect in the main SPLS analysis, dominated by age. Unlike the SPLS analysis, however, the remaining (non-sparse) weights don't focus on drinking behavior but instead on mood-related attributes that may co-vary with age. Thus, lower age is associated with irritability ("I got grumpy and cross easily", "I got angry easily"), and more interpersonal forms of anxiety, e.g. "I get anxious when meeting people", "I tend to avoid eye contact", "someone in my family hated me", "I worried what other people thought about me", "people were against me", "others do things more easily than I could". Conversely, older age is associated with more anhedonic or asocial feelings, e.g. "I do not have an expressive and lively way of speaking", feeling "hopeless", "I rarely laugh and smile", "I feel bad or guilty", "I am mostly quiet when with other people", "I am poor at returning social courtesies and

gestures". However, opposite ends of the effect also contain similar forms of low mood (e.g. "hard to get to sleep", "bad dreams", etc), and similar interpersonal actions (e.g. "you enjoy bothering or hurting other people", "you tease or make fun of other people" and "you are concerned about the feelings of others", "you share your things with other people without being asked"). These may just be due to chance differences in how children of different age rated these questionnaire items.

The brain weights of this brain-behavior relationship are similar to the main SPLS analysis in a way that the selected brain regions by SPLS were the ones that had ~1 weight values, which correspond to the highest value in the weight scale due to normalization. These included frontoparietotemporal cortical regions such the orbitofrontal cortex, mid- and posterior cingulate and medial orbital cortices, inferior parietal cortex, dorsolateral prefrontal cortex, right inferior frontal gyrus and middle temporal gyri. The brain weights are summarized in Supplemental Table S8.

The behavioral weights of the second brain-behavior relationship identified by KCCA analysis are very similar to the depression-related effects from both SPLS analyses i.e., excluding or including age in the confounds. All seven items from the depression-related effect in the former SPLS analysis reappear in this effect, which also shares 26 of its top 40 items with the depression-related effect from the latter SPLS analysis. As before, almost all the items relate to suicidality, depressive cognitions including low self-esteem, anhedonia and loss of motivation.

Again, the brain weights of this effect are similar to the main SPLS analysis in a way that the selected brain regions by SPLS were the ones that had large weights. These included hippocampus, parahippocampal gyrus, insula, and subcortical regions such as amygdala, pallidum and putamen. The brain weights are summarized in Supplemental Table S8.

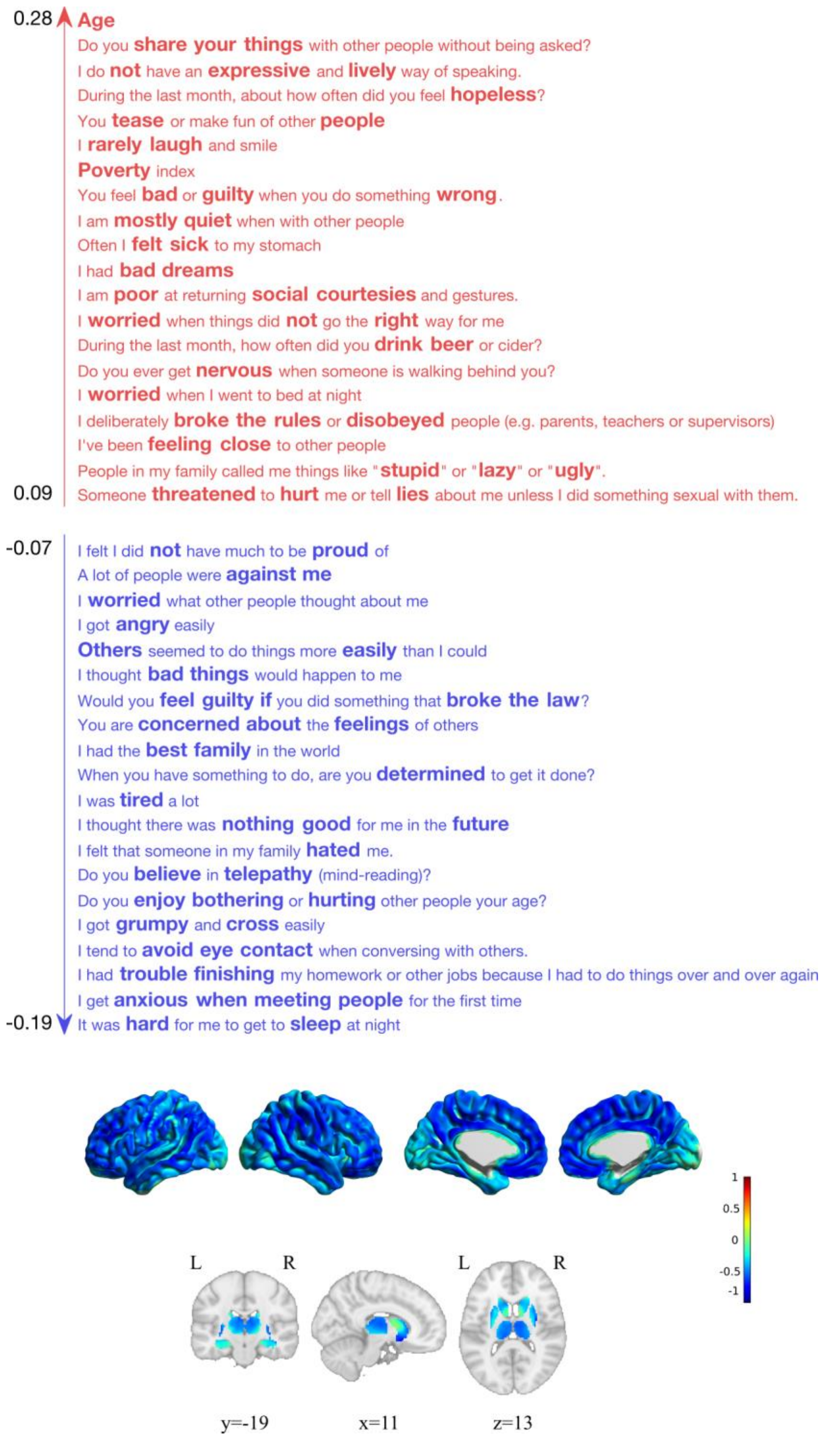

Supplemental Figure S7. Brain and behaviour weights of the first brain-behavior relationship identified by kernel canonical correlation analysis. The results are displayed for the data split that represents the best combination of generalizability (measured by the holdout correlation) and stability (measured by the average similarity of weights across the splits). Only the top 20 positive and top 20 negative behavioral weights are displayed. The brain voxels are colour coded by weight, normalized for visualization purposes and displayed on MNI152 template for all cortical and subcortical regions.

To quantitatively assess the similarity of the results, we calculated Pearson's correlation between the brain and behavioral weights/scores of this analysis and the main SPLS analysis. To account for the sparsity of the weights, we used only the corresponding weight vectors of the two methods restricting them to the same length as the sparse (SPLS) solution. For the first effect, the similarity between the brain and the behavioral weights of the two methods are $r_{brain} = 0.3359$ and $r_{behavior} = 0.9182$, respectively; the similarity between the brain and the behavioral scores of the two methods are $r_{brain} = 0.9495$ and $r_{behavior} = 0.5874$, respectively. We note that the very high value for the similarity of the behavioral weights is probably because of the main SPLS analysis identified a very sparse solution (including 8 variables in the model). For the second effect, the similarity between the brain and the behavioral weights of the two methods are $r_{brain} = 0.7468$ and $r_{behavior} = 0.7363$, respectively; the similarity between the brain and the behavioral scores of the two methods are $r_{brain} = 0.7268$ and $r_{behavior} = 0.8010$, respectively.

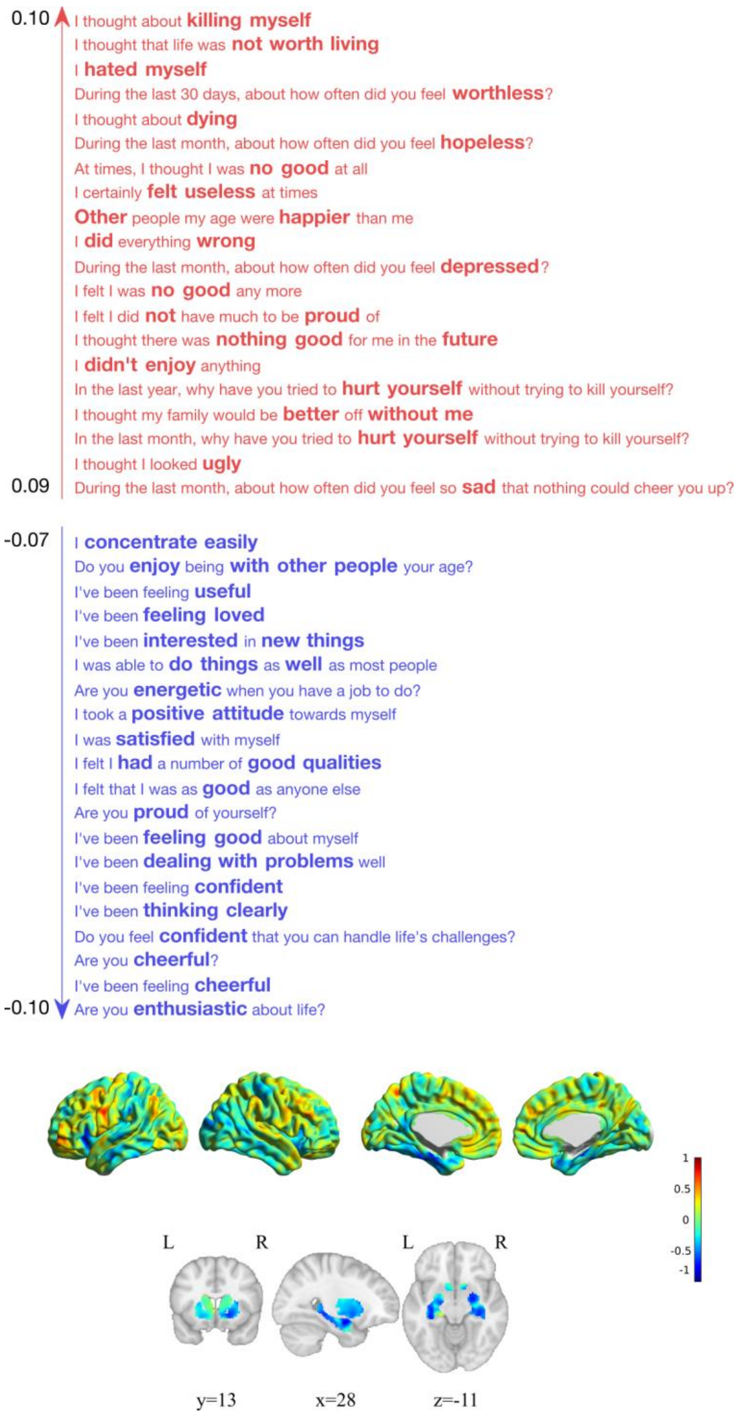

Supplemental Figure S8. Brain and behaviour weights of the second brain-behavior relationship identified by kernel canonical correlation analysis. The results are displayed for the data split that represents the best combination of generalizability (measured by the holdout correlation) and stability (measured by the average similarity of weights across the splits). Only the top 20 positive and top 20 negative behavioral weights are displayed. The brain voxels are colour coded by weight, normalized for visualization purposes and displayed on MNI152 template for all cortical and subcortical regions.

Supplemental Table S8. Summary of the brain weights in the kernel canonical correlation analysis for the data split that represents the best combination of generalizability (measured by the holdout correlation) and stability (measured by the average similarity of weights across the splits). The brain weights are summarized by the top 20 brain regions using the Automated Anatomical Labeling atlas (43). L, Left; R, Right

Brain regions	Average weight	Number of voxels
**First associative effect**		
L gyrus rectus	-0.0031	850
L middle cingulate & paracingulate gyri	-0.0030	1928
L lateral orbital gyrus	-0.0029	197
L superior frontal gyrus (medial orbital part)	-0.0029	714
R lateral orbital gyrus	-0.0028	185
R gyrus rectus	-0.0028	742
R medial orbital gyrus	-0.0028	621
R middle cingulate & paracingulate gyri	-0.0027	2182
L olfactory cortex	-0.0027	280
R anterior orbital gyrus	-0.0027	648
R superior frontal gyrus (medial orbital part)	-0.0027	856
L medial orbital gyrus	-0.0027	550
L superior frontal gyrus (medial part)	-0.0026	2875
R posterior orbital gyrus	-0.0026	561
R olfactory cortex	-0.0026	289
R superior frontal gyrus (medial part)	-0.0025	2115
R inferior parietal gyrus (excluding supramarginal and angular gyri)	-0.0025	1310
L anterior cingulate & paracingulate gyri	-0.0025	1400
L posterior orbital gyrus	-0.0025	567
L supplementary motor area	-0.0024	2035
**Second associative effect**		
L hippocampus	-0.0047	930
R amygdala	-0.0042	248
R hippocampus	-0.0040	945
R putamen	-0.0036	1064
R parahippocampal gyrus	-0.0035	1132
L parahippocampal gyrus	-0.0033	972
L amygdala	-0.0031	220
R pallidum	-0.0028	280
L fusiform gyrus	-0.0028	2307
L insula	-0.0026	1842
L anterior orbital gyrus	0.0026	443
Vermis (part 7)	0.0025	194
R heschl's gyrus	0.0024	245
L putamen	-0.0020	1009
L precentral gyrus	0.0020	3319

Vermis (part 10)	-0.0018	100
L cerebellum (part 8)	-0.0016	1840
L lateral orbital gyrus	0.0016	197
L superior frontal gyrus (medial orbital part)	0.0016	714
R insula	-0.0015	1770

Supplemental References

1. Shawe-Taylor J, Cristianini N (2004): *Kernel Methods for Pattern Analysis*. Cambridge: Cambridge University Press.

2. Hardoon DR, Szedmak S, Shawe-Taylor J (2004): Canonical correlation analysis: An overview with application to learning methods. *Neural Comput*. 16: 2639–2664.

3. Witten DM, Tibshirani R, Hastie T (2009): A penalized matrix decomposition, with applications to sparse principal components and canonical correlation analysis. *Biostatistics*. 10: 515–34.

4. Zou H, Hastie T (2005): Regularization and variable selection via the elastic net. *J R Stat Soc Ser B Stat Methodol*. 67: 301–320.

5. Rosa MJ, Mehta MA, Pich EM, Risterucci C, Zelaya F, Reinders AATS, *et al.* (2015): Estimating multivariate similarity between neuroimaging datasets with sparse canonical correlation analysis: an application to perfusion imaging. *Front Neurosci*. 9: 366.

6. Moser DA, Doucet GE, Lee WH, Rasgon A, Krinsky H, Leibu E, *et al.* (2018): Multivariate associations among behavioral, clinical, and multimodal imaging phenotypes in patients with psychosis. *JAMA Psychiatry*. 75: 386–395.

7. Moser DA, Doucet GE, Ing A, Dima D, Schumann G, Bilder RM, Frangou S (2018): An integrated brain–behavior model for working memory. *Mol Psychiatry*. 23: 1974–1980.

8. Wang H-T, Poerio G, Murphy C, Bzdok D, Jefferies E, Smallwood J (2018): Dimensions of Experience: Exploring the Heterogeneity of the Wandering Mind. *Psychol Sci*. 29: 56–71.

9. Wang HT, Bzdok D, Margulies D, Craddock C, Milham M, Jefferies E, Smallwood J (2018): Patterns of thought: Population variation in the associations between large-scale network organisation and self-reported experiences at rest. *Neuroimage*. 176: 518–527.

10. Lee WH, Moser DA, Ing A, Doucet GE, Frangou S (2019): Behavioral and Health Correlates of Resting-State Metastability in the Human Connectome Project. *Brain Topogr*. 32: 80–86.

11. Monteiro JM, Rao A, Ashburner J, Shawe-Taylor J, Mourão-Miranda J (2015): Multivariate effect ranking via adaptive sparse PLS. *2015 Int Work Pattern Recognit NeuroImaging*. IEEE, pp 25–28.

12. Monteiro JM, Rao A, Shawe-Taylor J, Mourão-Miranda J (2016): A multiple hold-out framework for Sparse Partial Least Squares. *J Neurosci Methods*. 271: 182–194.

13. Leonenko G, Di Florio A, Allardyce J, Forty L, Knott S, Jones L, *et al.* (2018): A

data-driven investigation of relationships between bipolar psychotic symptoms and schizophrenia genome-wide significant genetic loci. *Am J Med Genet Part B Neuropsychiatr Genet*. 177: 468–475.

14. Krishnan A, Williams LJ, McIntosh AR, Abdi H (2011): Partial Least Squares (PLS) methods for neuroimaging: A tutorial and review. *Neuroimage*. 56: 455–475.

15. Wold S, Sjöström M, Eriksson L (2001): PLS-regression: a basic tool of chemometrics. *Chemom Intell Lab Syst*. 58: 109–130.

16. Wegelin JA (2000): *A survey on Partial Least Squares (PLS) methods, with emphasis on the two-block case*. .

17. Rosipal R, Krämer N (2006): Overview and Recent Advances in Partial Least Squares. In: Saunders C, Grobelnik M, Gunn S, Shawe-Taylor J, editors. *Subspace, Latent Struct Featur Sel*. Berlin, Heidelberg: Springer Berlin Heidelberg, pp 34–51.

18. Abdi H (2010): Partial least squares regression and projection on latent structure regression (PLS Regression). *Wiley Interdiscip Rev Comput Stat*. 2: 97–106.

19. Baldassarre L, Pontil M, Mourão-Miranda J (2017): Sparsity Is Better with Stability: Combining Accuracy and Stability for Model Selection in Brain Decoding. *Front Neurosci*. 11.

20. Harris PA, Taylor R, Thielke R, Payne J, Gonzalez N, Conde JG (2009): Research electronic data capture (REDCap)-A metadata-driven methodology and workflow process for providing translational research informatics support. *J Biomed Inform*. 42: 377–381.

21. Kiddle B, Inkster B, Prabhu G, Moutoussis M, Whitaker KJ, Bullmore ET, *et al.* (2018): Cohort Profile: The NSPN 2400 Cohort: a developmental sample supporting the Wellcome Trust NeuroScience in Psychiatry Network. *Int J Epidemiol*. 47: 18-19g.

22. Frick P, Hare R (2001): *The Antisocial Process Screening Device*. Toronto, Ontario, Canada: Multi-Health Systems.

23. Patton JH, Stanford MS, Barratt ES (1995): Factor structure of the Barratt impulsiveness scale. *J Clin Psychol*. 51: 768–74.

24. Lahey BB, Applegate B, Chronis AM, Jones HA, Williams SH, Loney J, Waldman ID (2008): Psychometric characteristics of a measure of emotional dispositions developed to test a developmental propensity model of conduct disorder. *J Clin Child Adolesc Psychol*. 37: 794–807.

25. Fink LA, Bernstein D, Handelsman L, Foote J, Lovejoy M (1995): Initial reliability and validity of the childhood trauma interview: a new multidimensional measure of childhood interpersonal trauma. *Am J Psychiatry*. 152: 1329–35.

26. Essau CA, Sasagawa S, Frick PJ (2006): Callous-unemotional traits in a community sample of adolescents. *Assessment*. 13: 454–69.

27. Kessler RC, Andrews G, Colpe LJ, Hiripi E, Mroczek DK, Normand S-LT, *et al.* (2002): Short screening scales to monitor population prevalences and trends in non-specific psychological distress. *Psychol Med*. 32: 959–76.

28. Bamber D, Tamplin A, Park RJ, Kyte ZA, Goodyer IM (2002): Development of a short leyton obsessional inventory for children and adolescents. *J Am Acad Child Adolesc Psychiatry*. 41: 1246–52.

29. Costello EJ, Angold A (1988): Scales to assess child and adolescent depression: checklists, screens, and nets. *J Am Acad Child Adolesc Psychiatry*. 27: 726–737.

30. Reynolds CR, Richmond BO (1997): What I think and feel: a revised measure of Children's Manifest Anxiety. *J Abnorm Child Psychol*. 25: 15–20.

31. Rosenberg M (1965): *Society and the adolescent self-image.* (Vol. 148), Princeton: Princeton University Press.

32. Raine A (1991): The SPQ: a scale for the assessment of schizotypal personality based on DSM-III-R criteria. *Schizophr Bull*. 17: 555–64.

33. Axelrod BN (2002): Validity of the Wechsler abbreviated scale of intelligence and other very short forms of estimating intellectual functioning. *Assessment*. 9: 17–23.

34. Tennant R, Hiller L, Fishwick R, Platt S, Joseph S, Weich S, *et al.* (2007): The Warwick-Edinburgh Mental Well-being Scale (WEMWBS): development and UK validation. *Health Qual Life Outcomes*. 5: 63.

35. Weiskopf N, Suckling J, Williams G, Correia MM, Inkster B, Tait R, *et al.* (2013): Quantitative multi-parameter mapping of R1, PD*, MT, and R2* at 3T: a multi-center validation. *Front Neurosci*. 7: 1–11.

36. Tabelow K, Balteau E, Ashburner J, Callaghan MF, Draganski B, Helms G, *et al.* (2019): hMRI – A toolbox for quantitative MRI in neuroscience and clinical research. *Neuroimage*. 194: 191–210.

37. Helms G, Draganski B, Frackowiak R, Ashburner J, Weiskopf N (2009): Improved segmentation of deep brain grey matter structures using magnetization transfer (MT) parameter maps. *Neuroimage*. 47: 194–198.

38. Ashburner J, Friston KJ (2005): Unified segmentation. *Neuroimage*. 26: 839–851.

39. Ashburner J, Friston KJ (2011): Diffeomorphic registration using geodesic shooting and Gauss-Newton optimisation. *Neuroimage*. 55: 954–967.

40. Smith SM, Jenkinson M, Woolrich MW, Beckmann CF, Behrens TEJ, Johansen-Berg H, *et al.* (2004): Advances in functional and structural MR image analysis and implementation as FSL. *Neuroimage*. 23 Suppl 1: S208-19.

41. Abraham A, Pedregosa F, Eickenberg M, Gervais P, Mueller A, Kossaifi J, *et al.* (2014): Machine learning for neuroimaging with scikit-learn. *Front Neuroinform*. 8: 14.

42. Xia M, Wang J, He Y (2013): BrainNet Viewer: a network visualization tool for human brain connectomics. *PLoS One*. 8: e68910.

43. Rolls ET, Joliot M, Tzourio-Mazoyer N (2015): Implementation of a new parcellation of the orbitofrontal cortex in the automated anatomical labeling atlas. *Neuroimage*. 122: 1–5.
